# Topoisomerase II beta interacts with cohesin and CTCF at topological domain borders

**DOI:** 10.1186/s13059-016-1043-8

**Published:** 2016-08-31

**Authors:** Liis Uusküla-Reimand, Huayun Hou, Payman Samavarchi-Tehrani, Matteo Vietri Rudan, Minggao Liang, Alejandra Medina-Rivera, Hisham Mohammed, Dominic Schmidt, Petra Schwalie, Edwin J. Young, Jüri Reimand, Suzana Hadjur, Anne-Claude Gingras, Michael D. Wilson

**Affiliations:** 1 Genetics and Genome Biology Program, SickKids Research Institute, Toronto, ON Canada; 2 Department of Gene Technology, Tallinn University of Technology, Tallinn, Estonia; 3 Department of Molecular Genetics, University of Toronto, Toronto, ON Canada; 4 Lunenfeld-Tanenbaum Research Institute, Mount Sinai Hospital, Toronto, ON Canada; 5 Research Department of Cancer Biology, Cancer Institute, University College London, London, UK; 6 Cancer Research UK, Cambridge Institute, University of Cambridge, Cambridge, UK; 7 European Molecular Biology Laboratory, European Bioinformatics Institute, Cambridge, UK; 8 Ontario Institute for Cancer Research, Toronto, ON Canada; 9 Department of Medical Biophysics, University of Toronto, Toronto, ON Canada; 10 Present address: The Babraham Institute, Cambridge, UK; 11 Present address: Syncona Partners LLP, London, UK; 12 Present address: Laboratory of Systems Biology and Genetics, Lausanne, Switzerland; 13 Present address: International Laboratory for Research in Human Genomics, Universidad Nacional Autónoma de México, Juriquilla, Querétaro Mexico

**Keywords:** Topoisomerase II beta, CTCF, Cohesin, BioID, ChIP-seq, ChIP-exo, Hi-C, Comparative genomics, Proteomics, Topological associated domains, DNA supercoiling, Genome organization

## Abstract

**Background:**

Type II DNA topoisomerases (TOP2) regulate DNA topology by generating transient double stranded breaks during replication and transcription. Topoisomerase II beta (TOP2B) facilitates rapid gene expression and functions at the later stages of development and differentiation. To gain new insight into the genome biology of TOP2B, we used proteomics (BioID), chromatin immunoprecipitation, and high-throughput chromosome conformation capture (Hi-C) to identify novel proximal TOP2B protein interactions and characterize the genomic landscape of TOP2B binding at base pair resolution.

**Results:**

Our human TOP2B proximal protein interaction network included members of the cohesin complex and nucleolar proteins associated with rDNA biology. TOP2B associates with DNase I hypersensitivity sites, allele-specific transcription factor (TF) binding, and evolutionarily conserved TF binding sites on the mouse genome. Approximately half of all CTCF/cohesion-bound regions coincided with TOP2B binding. Base pair resolution ChIP-exo mapping of TOP2B, CTCF, and cohesin sites revealed a striking structural ordering of these proteins along the genome relative to the CTCF motif. These ordered TOP2B-CTCF-cohesin sites flank the boundaries of topologically associating domains (TADs) with TOP2B positioned externally and cohesin internally to the domain loop.

**Conclusions:**

TOP2B is positioned to solve topological problems at diverse cis-regulatory elements and its occupancy is a highly ordered and prevalent feature of CTCF/cohesin binding sites that flank TADs.

**Electronic supplementary material:**

The online version of this article (doi:10.1186/s13059-016-1043-8) contains supplementary material, which is available to authorized users.

## Background

The type II topoisomerase (TOP2) enzymes resolve DNA topology problems in core biological processes such as transcription, replication, recombination, DNA repair, chromatin remodeling, chromosome condensation, and segregation [1–3]. TOP2 enzymes catalyze and rejoin transient DNA double-stranded breaks (DSB) by allowing one of the duplex DNA strands to pass through the other [1–3]. Vertebrates possess two TOP2 genes, *TOP2A* and *TOP2B*, that originate from an ancestral gene duplication event [4, 5]. TOP2A and TOP2B are not functionally redundant despite their structural and catalytic similarities [6]. TOP2A is expressed in proliferating cells [7, 8] and knocking out *Top2a* in mice leads to defects in nuclear division and early embryonic lethality [9–11]. In contrast, TOP2B is ubiquitously expressed and is upregulated during cellular differentiation [7].

The full knockout of *Top2b* in mice leads to perinatal lethality mediated by defects in neuronal differentiation [12]. Conditional *Top2b* mouse knockout studies have demonstrated TOP2B’s importance during retinal development [13] and ovulation [14]. Studies using TOP2 poisons have implicated TOP2B in spermatogenesis [15–17] and lymphocyte activation [18]. In contrast to these functional insights, the conditional ablation of TOP2B in the adult heart resulted in few significant gene expression changes [19]. Despite the growing number of tissues and developmental processes that require TOP2B, the mechanisms by which this ubiquitous protein facilitates tissue-specific developmental processes are still not well understood.

It has been proposed that TOP2B’s role in development involves the activation or repression of specific developmental genes [20, 21]. Human TOP2B is required for the activation of hormone sensitive genes through the generation of transient double-stranded DNA breaks at the promoter region [20, 22]. Most recently, TOP2B-generated DSBs have been shown to be essential for the activation of early response genes by neurotransmitters [23]. Moreover, TOP2B has also been implicated in the expression of long genes, presumably through its ability to resolve positive supercoiling that arises during transcription [24].

TOP2B is also actively studied in the context of cancer. For example, TOP2B-mediated cleavage occurs at known chromosomal breakpoints in prostate cancer [25] and has been observed near translocation breakpoints in leukemia [26]. TOP2 proteins are prominent targets of many widely used chemotherapy agents including doxorubicin, etoposide, and mitoxantrone [27]. However, these chemotherapeutic agents can cause secondary malignancies in non-neoplastic tissues (reviewed in [28]). Whereas TOP2A is the intended target of these widely used chemotherapeutic agents, mechanistic studies in cell lines and animal models show that TOP2B-mediated DNA cleavage is an important player in treatment-related malignancies [19, 25, 29]. Intriguingly, heart-specific ablation of TOP2B significantly reduced the cardiotoxicity that normally occurs from doxorubicin treatment [19].

Identifying the protein–protein and protein–DNA interactions of TOP2B is essential for understanding its roles in development, transcription, and cancer. Here we report a comprehensive proximal protein interaction network for TOP2B that includes several members of the cohesin complex. Using ChIP-seq and ChIP-exo in combination with high-throughput chromosome conformation capture (Hi-C) data, we find that TOP2B interacts with CTCF and the cohesin complex with a distinct spatial organization at the borders of long-range chromosomal domain structures.

## Results

### TOP2B interacts with CTCF and the cohesin complex

**Fig. 1:**
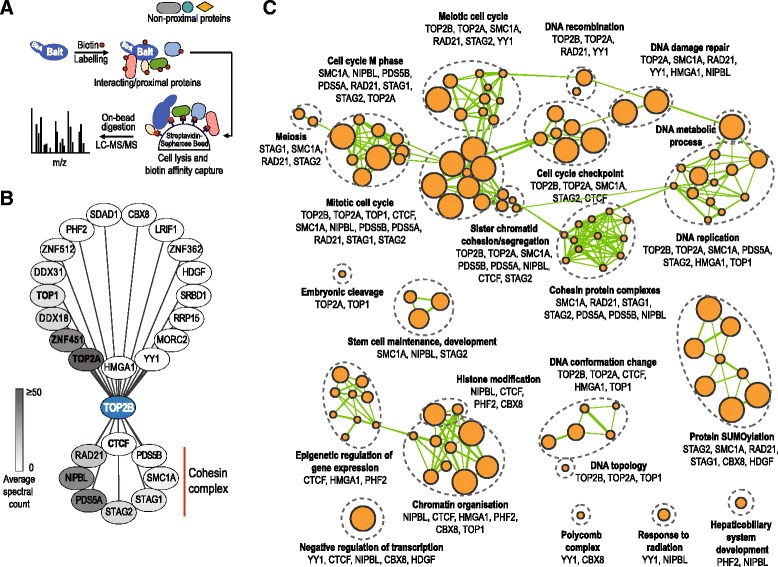
TOP2B interactome. **a** Overview of BioID method [31, 32]. In BioID, mutated *Escherichia coli* biotin protein ligase BirA* is fused with the protein of interest (bait) resulting in vivo biotinylation of proximal and interacting proteins of the bait protein. Subsequent to cell lysis, biotinylated proteins are captured by streptavidin and identified by mass spectrometry. **b** High-confidence interaction partners of TOP2B (*n* = 25, FDR ≤5 %) are visualized as a network displaying the average spectral counts of a given prey protein. Known TOP2B interacting proteins are shown in *bold*. Cohesin complex proteins (RAD21, NIPBL, PDS5A, PDS5B, STAG1, STAG2, SMC1A) and CTCF are indicated. **c** *Enrichment Map network visualization* of statistically over-represented biological processes in the TOP2B interactome (FDR *q* <0.05). Nodes in the network represent enriched processes and pathways that are grouped according to prevalent functional themes. Node size is proportional to the total number of genes within each gene set. Genes corresponding to interacting proteins are shown

We first set out to characterize a TOP2B protein–protein interaction network. Topoisomerases are large and relatively insoluble proteins [30] that present challenges for classical affinity purification. To circumvent these problems, we employed BioID, an in vivo interaction mapping approach in which a bait protein of interest is fused to a modified biotin ligase enzyme (BirA*) that leads to covalent biotinylation of proteins in close proximity to the expressed proteins (Fig. 1a). Biotinylated proteins can be recovered under high stringency lysis and washes conditions (detergents, salt, DNA shearing) that would not normally be compatible with native purification (Fig. 1a). BioID also provides increased sensitivity over standard purifications by enabling recovery of both the direct physical interaction partners of the protein of interest as well as its vicinal proteins in live cells and has been used previously to detect novel chromatin associated complexes [31, 32]).

We performed BioID in HeLa cells with a TOP2B bait protein tagged with a N-terminal BirA*-FLAG tag (*n* = 6). Control experiments involved parental cells (no BirA*), a BirA*-FLAG fused to green fluorescent protein (GFP-bait), and a BirA*-FLAG tag fused to a nuclear localization signal (NLS-bait) (see “Methods”). Mass spectrometry revealed 737 proteins with at least two unique peptides for the TOP2B bait (Additional file 1). We detected 25 high confidence interaction partners for TOP2B (SAINT Bayesian false discovery rate (FDR) ≤5 %); Fig. 1b, Additional file 1).

Supporting the sensitivity of the BioID method, we recovered several previously known interaction partners of TOP2B: TOP2A forms active heterodimers with TOP2B in HeLa cells [33]; TOP1 forms the DNA synthesome complex with TOP2B during DNA replication [34]; CTCF has been previously shown to interact with TOP2B in human breast cancer cell lines [35]; and ZNF451, a Smad3/4 transcriptional co-repressor [36] has been previously co-purified with TOP2B using tandem affinity purification mass-spectrometry [37]. Although we did not detect significant interactions with HMGB1 (FDR = 17 %) implicated in TOP2B-mediated transcriptional regulation [22], we identified a canonical high mobility group (HMG) family member HMGA1 and an HMG-like protein HDGF [38], as well as additional 19 novel TOP2B interacting proteins (FDR ≤5 %; Fig. 1b). TOP2B is known to localize to the nucleolus [39] and our BioID experiments revealed novel interactions of TOP2B with known nucleolar proteins involved in rDNA gene regulation (DDX18, DDX31, SDAD1, RRP15). Also among the novel TOP2B interactions were several cohesin subunits (RAD21, STAG1, STAG2, SMC1A) and cohesin-associated proteins (NIPBL, PDS5A, PDS5B; Fig. 1b, c, Additional file 1). The specificity of the CTCF and cohesin enrichments in TOP2B over the controls were confirmed by repeating the biotin labeling and capture experiments followed by western blot using antibodies against RAD21 and CTCF (Additional file 2: Figure S1).

### TOP2B is bound to critical points of genome control

To investigate whether the physical interactions of TOP2B are also reflected at the genomic level, we profiled DNA occupancy of TOP2B in primary mouse liver cells using chromatin immunoprecipitation followed by DNA sequencing (ChIP-seq). The mouse liver is an optimal TOP2B-expressing tissue for our in vivo experiments as it provides an abundant and relatively homogenous source of non-dividing cells and is an actively used model for mammalian gene regulation with a wealth of functional genomic datasets available [40–42] (Additional file 2: Table S1 and Additional file 3).

**Fig. 2:**
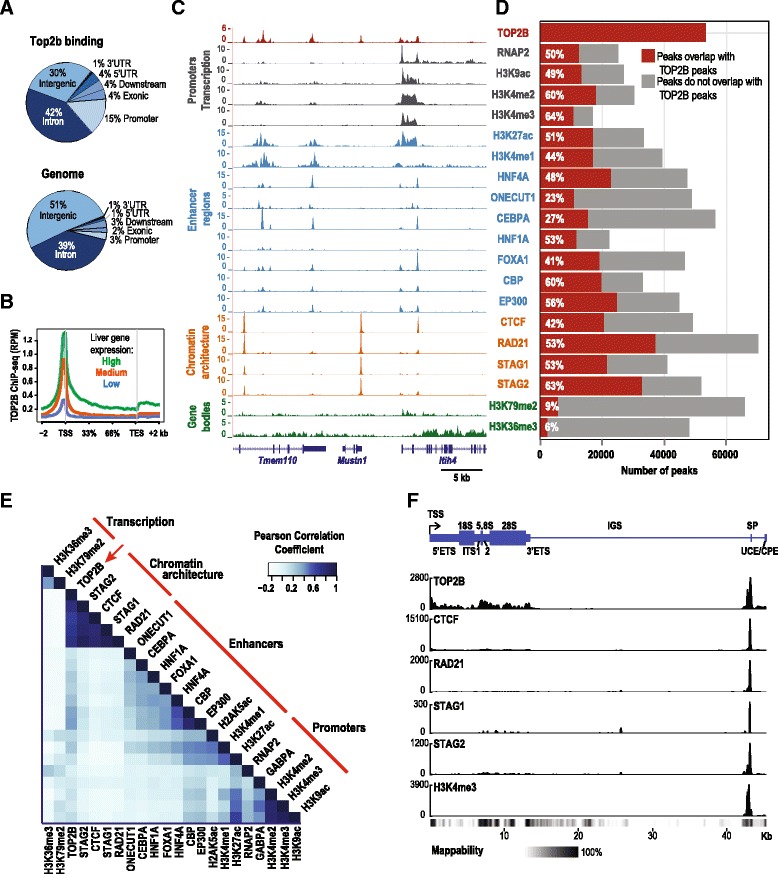
Genomic annotation of TOP2B binding sites in mouse liver. **a** Genome-wide distribution of TOP2B binding sites compared to the general distribution of mouse genomic regions. Promoter regions were defined as 3 kb upstream from transcription start site (TSS); downstream is defined as 3 kb from transcription end site (TES). **b** Aggregate TOP2B liver ChIP-seq read density (*x-axis*) across liver-expressed genes separated into high, medium, and low expression categories (*y-axis*). ChIP-seq read density is shown as reads per million mapped reads (RPM). **c** Genome browser view for ChIP-seq signal tracks for TOP2B and a variety of liver-enriched TFs, members of the cohesin complex and histone modifications (*y-axis*, RPM). **d** Fraction of peaks for various factors that overlap TOP2B peaks (*red bars*) (*p* values <10^–16^). **e** Hierarchical clustering of genome-wide binding intensities (reads per kilobase per million mapped reads (RPKM)) for TOP2B and a variety of factors. Color intensity represents pair-wise Pearson correlation coefficients. *Red lines* indicate the main clusters: actively transcribed regions (i.e. H3K36me3, H3K79me2), genome architectural regions (i.e. RAD21), enhancers (i.e. ONECUT1), and promoters (i.e. H3K4me3). **f** TOP2B localizes to rDNA loci in mouse liver. The *top panel* shows a *schematic representation* of a single mouse rDNA repeat relative to the transcription start site of the rDNA repeat (*x-axis*; based off of GenBank BK000964). Normalized ChIP-seq read counts of TOP2B, CTCF, RAD21, STAG1, STAG2, and H3K4me3 are shown on the *y-axis*. rDNA mappability track from Zentner GE et al. [78] is plotted as a *heatmap* below the genome tracks with *black* representing bases that are 100 % mappable. The TSS is labeled with an *arrow*. The 18S, 5.8S, and 28S coding regions are shown as *large rectangles*. The external transcribed spacer; (ETS), internal transcribed spacer (ITS), spacer promoter (SP), upstream control element (UCE), and core promoter element (CPE), are shown as *narrow rectangles*. The intergenic spacer (IGS) is shown as a *thin line*

As expected, we found an enrichment of TOP2B binding at gene promoter regions (*p* <10^–16^, Fig. 2a) [20–22, 25, 43] and at highly expressed genes (Fig. 2b). TOP2B binding also coincided with histone marks of active transcription (H3K4me3, H3K9ac) and enhancers (H3K27ac, H3K4me1), as well as binding sites of liver-specific transcription factors (TFs) FOXA1, ONECUT1, HNF1A, HNF4A, and CEBPA (*q* < 10^–3^, Fig. 2c–e) [40, 41]. Supporting the proximal protein interaction network obtained from human cells, TOP2B co-localized with ChIP-seq binding sites for CTCF and cohesin subunits RAD21, STAG1, and STAG2 in mouse liver [40] (Fig. 2e).

To look for evidence of TOP2B interaction at rDNA loci we aligned TOP2B ChIP-seq data to a single mouse rDNA repeat. We observed a clear localization of TOP2B to the spacer promoter region as well as along the length of the rDNA transcribed region. At the spacer promoter, we also detected a substantial overlap of TOP2B, CTCF, and cohesin complex members (Fig. 2f).

**Fig. 3:**
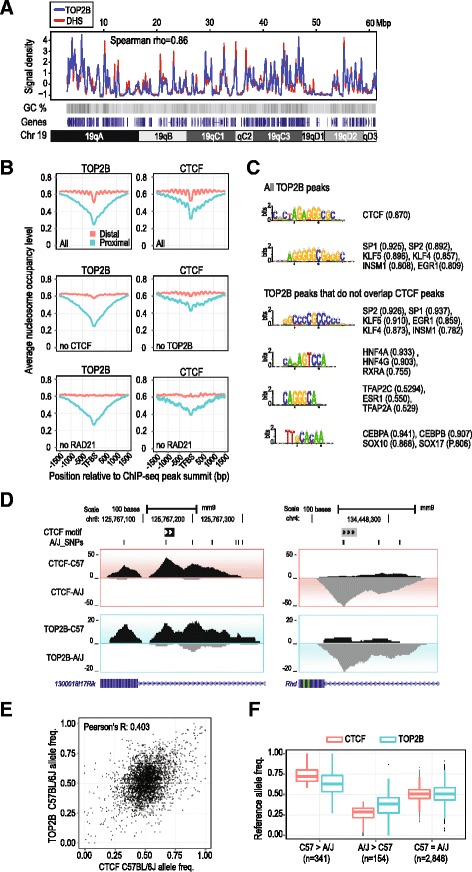
TOP2B preferentially interacts with open chromatin. **a** Correlation of TOP2B ChIP-seq and DNase I hypersensitivity sequencing signal densities across mouse chromosome 19 (Spearman rho = 0.86). Gene density and GC content are also shown. **b** Nucleosome occupancy profiles for all TOP2B and CTCF peaks (All) centered on the peak summit are shown for proximal (≤1 kb from TSS, *red lines*) and distal peaks (>1 kb from TSS, *blue lines*). Profiles for TOP2B peaks not overlapping CTCF peaks (no CTCF), CTCF peaks not overlapping TOP2B peaks (no TOP2B), TOP2B and CTCF peaks not overlapping RAD21 peaks (no RAD21) are also shown. **c** De novo motif discovery using all TOP2B peaks (50 bp upstream/downstream of the summit) and TOP2B peaks not overlapping CTCF peaks. **d** Genome browser view of example CTCF bound regions that show allele-specific bias towards the C57BL/6J genome (*left panel*) and A/J genome (*right panel*) (*y-axis*, number of allelic reads). **e** Correlation of C57BL/6J allele frequencies for CTCF (*x-axis*) versus TOP2B (*y-axis*) at TOP2B/CTCF co-occupied regions. **f** TOP2B (*blue*) and CTCF (*red*) C57BL/6J allele frequencies (*blue*) at TOP2B/CTCF co-occupied regions, categorized based on CTCF allelic binding preference (see “Methods”). C57 > A/J indicates sites with CTCF preference for the C57BL/6J allele, A/J > C57 indicates preference for the A/J allele, and C57 ~ A/J indicates sites with no significant allelic-specific bias. The TOP2B allelic frequencies for both C57BL/6J and A/J enriched CTCF binding sites were significantly different than the TOP2B allelic frequencies in C57 ~ A/J category (*p* <10^–6^; one-sided Wilcoxon rank-sum test)

Given its broad correlation with several actively regulated epigenetic marks and TF binding sites, we asked whether TOP2B’s occupancy at gene promoters, enhancers, and CTCF sites generally reflects its binding preference for open chromatin. Using deeply sequenced mouse liver DNase I hypersensitivity (DHS) data [44], we found a strong correlation of TOP2B binding and DHS signal profiles (Spearman *ρ* = 0.8, *p* <10^–16^; Fig. 3a, Additional file 2: Table S2). This correlation of DHS and TOP2B ChIP-seq signal was stronger than observed for any of the 20 factors we tested (Additional file 2: Table S2).

Consistent with its preference for DHS regions, we find that TOP2B occupancy is enriched at nucleosome-free regions delineated by MNase-seq experiments performed in mouse liver [45]. Specifically, nucleosome positioning relative to TOP2B peak summits found at proximal promoters (<1 kb from nearest TSS) is similar to what we observed for several TFs (Fig. 3b, Additional file 2: Figure S2). At distal TOP2B binding summits (>1 kb from nearest TSS), we found a periodic nucleosome occupancy pattern that closely resembled the nucleosome profiles around CTCF and RAD21 summits (Fig. 3b, Additional file 2: Figure S2). As CTCF strongly influences nucleosome positioning [46, 47], we analyzed nucleosome positioning around distal CTCF peak summits that did not overlap TOP2B peaks. The amplitude of periodic nucleosome occupancy around these non-TOP2B CTCF sites was clearly reduced suggesting that TOP2B occupancy is a biochemical feature of CTCF binding sites showing strong nucleosomal positioning (Fig. 3b).

### TOP2B DNA occupancy is influenced by TF binding

Next, we characterized the sequence properties of TOP2B bound regions using de novo motif discovery (see “Methods”). The most abundant motif recovered closely resembled the CTCF motif and was identified at ~17 % of TOP2B binding sites (Fig. 3c, Additional file 4). Two recent studies also identified an enrichment of CTCF motifs at TOP2B binding sites in mouse neurons [23] and in human MCF7 cells [48] demonstrating that CTCF motifs are a common feature of TOP2B occupied regions in multiple tissues. In addition, we repeated the de novo motif discovery after excluding joint binding sites of TOP2B and CTCF. We also recovered motifs similar to tissue-enriched factors HNF4A and CEBPA, as well as ESR1 which was previously reported as being enriched in MCF7 TOP2B ChIP-seq [48] data. These data collectively show that motifs of tissue-enriched TFs are also a common feature of TOP2B binding (Fig. 3c, Additional file 4).

To gain insight into whether changes in sequence specific TF binding correlate with the binding of TOP2B, we analyzed the allele-specific binding of CTCF, HNF4A, and TOP2B obtained from ChIP-seq experiments in livers from F1 mice (C57BL6/J female × A/J male) (Fig. 3d). We found that the ratio of allele-specific TOP2B ChIP-seq reads (shown as C57BL6/J allele frequency) correlates with the ratio of allele-specific CTCF ChIP-seq reads (*r* = 0.403, *p* <10^–16^) (Fig. 3e). We identified 495 CTCF/TOP2B co-bound sites with significant allele-specific bias of CTCF reads (binomial *p* value <0.05, see “Methods” for details) (Fig. 3f). At these sites, TOP2B and CTCF showed preference for the same allele and the allelic ratios of CTCF and TOP2B ChIP-seq reads were significantly skewed compared to CTCF/TOP2B sites with no allele specific CTCF binding (*p* <10^–16^, one-sided Wilcoxon rank sum test; Fig. 3f). Similarly, the allele specificity of TOP2B and HNF4A was also correlated at HNF4A/TOP2B bound sites (*r* = 0.546, *p* <10^–16^) (Additional file 2: Figure S3). In summary, although TOP2B has previously been suggested to have a DNA binding motif [49], we instead propose a model where TOP2B interacts with DNA that is actively bound by a variety of sequence-specific TFs without the need for specific motif recognition sequences.

### TOP2B co-localizes with evolutionarily conserved CTCF/cohesin binding sites

**Fig. 4:**
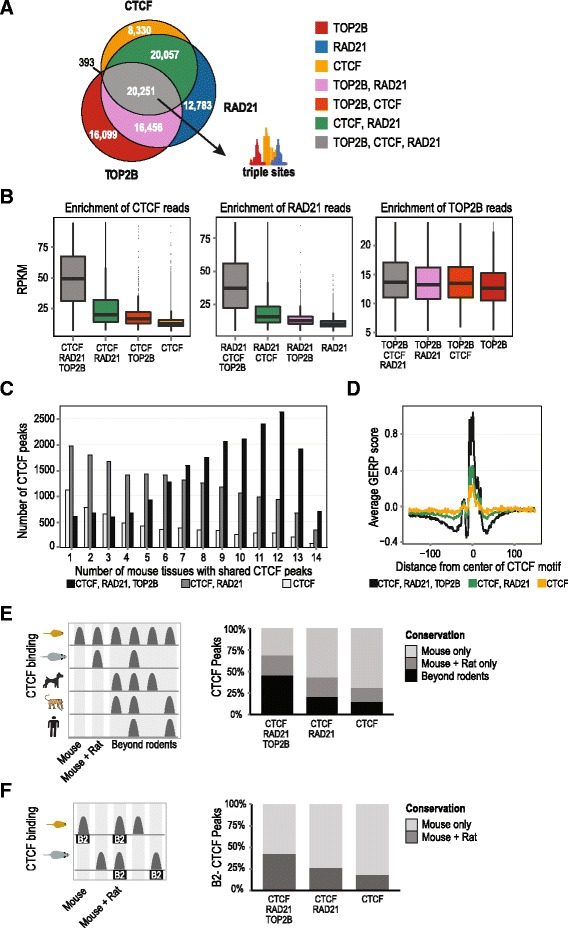
Genomic features of combinatorial TOP2B, CTCF, and RAD21 binding. **a** Overlap of TOP2B, CTCF, and RAD21 ChIP-seq binding regions defines seven different categories of peaks. **b** Comparison of CTCF, RAD21, and TOP2B ChIP-seq reads (RPKM) for each of the categories defined in (**a**). Outliers (>95th percentile) are not shown. **c** Pan-tissue analysis of CTCF binding for triple sites (*black*), double sites (*dark gray*), and CTCF-only sites (*white*). **d** DNA sequence constraint of triple (*black*), double (*green*), and CTCF only sites (*yellow*) as determined by average GERP score (*y-axis*). CTCF peaks were oriented based on the direction of CTCF motif (G-rich orientation is shown). **e** Cross-species comparison of mouse triple (*n* = 20,381), double (*n* = 20,049), and CTCF-only sites (*n* = 8301) with CTCF ChIP-seq peaks mapped in human, macaque, rat, and dog. *Stacked bar plots* show the proportion of peaks based on the degree of conservation (mouse only, mouse and rat only, and mouse plus one non-rodent (beyond rodents)). **f** Conservation of mouse triple (*n* = 1329), double (*n* = 6582), and singleton (*n* = 2957) CTCF sites that contain rodent-specific B2 SINE elements

To investigate whether CTCF and cohesin sites occupied by TOP2B possess unique biochemical and evolutionary features, we first explored the genomic co-occupancy of these proteins. TOP2B was found at approximately half of the CTCF/RAD21 sites (20,251 TOP2B, CTCF, and RAD21 triple sites versus 20,057 CTCF/RAD21 double sites (Fig. 4a). In contrast, we identified only 393 TOP2B/CTCF sites, indicating that TOP2B/CTCF interactions occur almost exclusively in the context of cohesin occupancy.

In order to gain insight into functional properties imparted directly or indirectly by TOP2B occupancy at CTCF/cohesin sites, we compared several genomic features between TOP2B/CTCF/RAD21 “triple sites” and CTCF/RAD21 “double sites.” Triple sites have significantly higher CTCF and RAD21 ChIP signal compared to CTCF/RAD21 double sites (fold change >2, one-sided Wilcoxon rank sum test, *p* <10^–16^; Fig. 4b). Triple sites are also more likely to be occupied by CTCF in multiple tissues. For example, 68 % of triple sites overlap with CTCF binding sites shared in seven or more tissues [41], in contrast to only 37 % of CTCF/RAD21 double sites (*p* <10^–16^, one-sided Fisher’s exact test; Fig. 4c).

Evolutionary conservation of gene regulatory regions is frequently used as a proxy for functional importance. We asked whether CTCF sites classified as triple sites in mouse were more evolutionarily conserved than CTCF/RAD21 double sites. Using genomic evolutionary rate profiling (GERP) [50] to measure DNA constraint, we found that triple sites were more conserved than CTCF/RAD21 double sites (Fig. 4d). We confirmed that the peak of DNA constraint over the region upstream of the CTCF core motif corresponds to the previously described CTCF upstream motif [42, 51]. We found that the upstream motif is present in a minority (~13 %) of our CTCF peaks, which is consistent with previously reported results [42, 51]. We observed that the “*core + upstream CTCF motif*” containing triple sites have a clear increase in DNA constraint at the upstream motif location compared to the “*CTCF core motif only*” triple sites (Additional file 2: Figure S4a). We also observed that HNF4A binding sites that co-occur with TOP2B binding sites show higher ChIP-seq signal and DNA constraint than sites without TOP2B binding (Additional file 2: Figure S4b–d).

We then asked whether TOP2B binding at CTCF/RAD21 binding sites corresponds to shared orthologous CTCF sites using CTCF ChIP-seq data previously ascertained for human, macaque, rat, and dog liver tissue [42]. We found that 45 % of CTCF peaks in triple sites were shared in at least one non-rodent species (see “Methods”) in contrast to 21 % of CTCF/RAD21 double sites (Fisher’s exact test, *p* <10^–16^; Fig. 4e; Additional file 2: Table S3). In addition, we also found that mouse HNF4A/TOP2B co-bound sites are more likely to be shared in a non-rodent species compared to HNF4A-only sites (26 % and 11 %, respectively, Fisher’s exact test, *p* <10^–16^; Additional file 2: Table S4; Additional file 2: Figure S4e).

Since rodent-specific transposable B2 SINE (Short Interspersed Element) sequences are a source of lineage-specific CTCF binding sites in rodent genomes [42, 52], we asked whether TOP2B binding enriched for recently evolved CTCF binding events derived from B2 elements that have been fixed in the rodent lineage. Indeed, we found that B2 SINE-derived CTCF sites that occur in the context of triple sites were more likely to be shared between mouse and rat (42 %) compared to CTCF/RAD21 double sites (26 %) (Fisher’s exact test, *p* <10^–16^; Fig. 4f; Additional file 2: Table S3). Thus TOP2B genomic occupancy appears to be a distinguishing feature of functionally relevant TF binding events.

### TOP2B and RAD21 are spatially organized around CTCF peaks

**Fig. 5:**
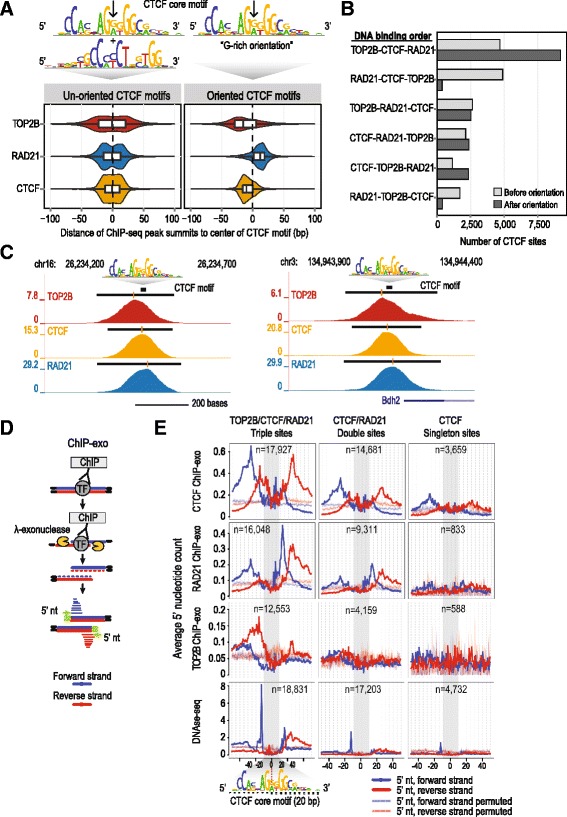
TOP2B, CTCF, and RAD21 binding is spatially organized. **a** Distribution of TOP2B, CTCF, and RAD21 ChIP-seq peak summits relative to the center of the CTCF core motif (*black arrow*). Distributions before (*left*) and after (*right*) ordering the CTCF peaks according to the orientation of the CTCF motif are shown and are significantly different (*p* value <10^–16^). **b** Number of triple sites with different TOP2B/CTCF/RAD21 binding orders relative to the center of the CTCF motif. Counts are shown before and after accounting for the CTCF motif orientation. **c** Genome browser tracks showing the ChIP-seq signal of TOP2B, CTCF, and RAD21 in two example regions on chromosomes 16 and 3. The *top track* shows the location of CTCF core motif, with the direction of it indicated by *white arrows. Black bars* above ChIP-seq signal tracks indicate peak locations with the *yellow lines* showing the location of peak summits. **d** Overview of ChIP-exo. After chromatin immunoprecipitation of cross-linked and sonicated chromatin, lambda exonuclease treatment selectively digests the 5′-phosphorylated strand of dsDNA. The resulting digestion leaves strand-specific footprints of the bound protein (labeled TF in the cartoon). DNA sequencing of these ChIP-exo fragments can reveal the border of where the DNA was protected by the bound protein. ChIP-exo results can be visualized by counting the pile-ups of the 5′ ends of DNA sequence reads (we have labeled the forward strand *blue* and the reverse strand *red*). **e** ChIP-exo protection signal profiles for CTCF, RAD21, and TOP2B ChIP-exo and DNase-seq (DGF) experiments. Average numbers of 5′ nucleotides of ChIP-exo reads (*blue*: forward strand; *red*: reverse strand) are plotted separately for triple, double, and singleton CTCF sites at each base pair (+/– 50 bp) relative to the center of the CTCF core motif. The number of regions used to generate each plot is labeled in the top right corner. The relative position of the CTCF core motif is shown

CTCF binds an asymmetric DNA motif with orientation dependent activities [40, 53–56]. To investigate the binding of TOP2B and RAD21 relative to CTCF, we characterized the relative order of TOP2B, CTCF, and RAD21 ChIP-seq binding sites in a ±100 bp region around the CTCF motif. Peak summits were used as proxies for binding sites and genomic distances between the binding sites and the center of CTCF motif were calculated, correcting for orientation of the motif (Fig. 5a). We found that TOP2B and RAD21 were spatially organized on opposite sides of the G-rich CTCF binding motif. TOP2B was positioned 5′ of the motif, with the median distance to the motif center being 15 bp, and RAD21 was positioned 3′ of the motif center, with a median distance of 12 bp. This spatial organization was apparent in the majority of triple sites (53.6 %, *p* <10^–16^, Fisher’s exact test) (Fig. 5b; Fig. 5c). This order also holds true when examining the binding of other cohesin complex subunits, STAG1 and STAG2 (Additional file 2: Figure S5a, b). Additionally, the motif of YY1 [57], an established co-factor of CTCF [58], and one of our significant TOP2B interacting proteins (Fig. 1b) was found 3′ of the G-rich CTCF motif (Additional file 2: Figure S5c). In contrast, no significant orientation bias was apparent in the binding of TOP2B and RAD21 around the binding motif of HNF4A (Additional file 2: Figure S5d).

To determine the precise spatial organization of triple sites, we performed ChIP-exo experiments [59] for TOP2B, CTCF, and RAD21 in mouse liver cells (Fig. 5d, e). ChIP-exo recovered the majority of CTCF peaks identified with ChIP-seq (76 %) (Additional file 2: Figure S6a). ChIP-exo for TOP2B and RAD21 recovered fewer peak regions than was obtained by ChIP-seq (16 % and 17 %, respectively (Additional file 2: Figure S6a)), as would be expected for factors that do not bind to specific DNA motifs. Importantly, the majority of the identified TOP2B and RAD21 ChIP-exo peaks overlapped with CTCF ChIP-exo peaks (82 % and 92 %, respectively) (Additional file 2: Figure S6b).

In order to obtain insights into the exonuclease protection signal of TOP2B and RAD21 relative to the CTCF motif at single base pair resolution, we plotted an average number of 5′ nucleotides of ChIP-exo reads aligned to each base pair around oriented CTCF motifs (Fig. 5e). We analyzed ChIP-exo signals separately at CTCF/RAD21 double sites and CTCF-only sites. Due to the correlation we observed for TOP2B binding and DNase I hypersensitivity signal, we also plotted mouse liver DNase I signal [41] alongside our ChIP-exo data. We recapitulated known exo-nuclease protection patterns for CTCF [51]. We also detected distinct patterns for TOP2B and RAD21. Relative to triple sites, the RAD21/CTCF double sites and CTCF-only sites showed less exo-nuclease protection signal and less DNase I hypersensitivity signal. Importantly, CTCF ChIP-exo protection profiles can be seen within our TOP2B ChIP-exo and RAD21 ChIP-exo protection profiles. These results indicate that, similar to what has been reported for CTCF and cohesin interactions [60], TOP2B can bind directly with DNA and also cross-link to DNA-bound CTCF.

Our ChIP-exo protection signal further confirmed the orientation-specific binding of RAD21 and TOP2B relative to CTCF (Fig. 5e). Specifically, ChIP-exo for RAD21 showed exonuclease protection at positions +13 to +26 (13–26 bp downstream) of the center of CTCF core motif (Fig. 5e). TOP2B ChIP-exo revealed a protection signal within positions –13 to –27 (13–27 bp upstream) of the center of the CTCF core motif. This TOP2B ChIP-exo protection signal was primarily observed on the reverse strand directly adjacent to the previously reported DNase I cleavage site located at –12 to –13 from the center of CTCF core motif that occurs on the positive strand (Fig. 5e; [61]). This raises the possibility that CTCF binding promotes DNA strand-specific interactions for TOP2B and DNase I enzymes.

Since the TOP2B ChIP-exo protection signal overlaps with the location of the upstream CTCF motif, which can be bound by CTCF zinc fingers 9–11 [51], we asked whether the presence of CTCF upstream motif would result in a distinct TOP2B ChIP-exo protection signal. We found that the upstream motif is present in a minority (~13 %) of our CTCF peaks, which is consistent with previously reported results [42, 51]. Our CTCF ChIP-exo profile within the “*core plus the upstream CTCF motif*” peaks showed the previously reported increase in ChIP-exo protection signal at positions –16 (reverse strand) and –25 (forward strand) [51] (Additional file 2: Figure S7). We also observed the previously reported decrease in DNase I signal at the –17 position [61]. Interestingly, we found that TOP2B ChIP-exo protection signal that we observed using all CTCF peaks (between positions –13 and –27) was less pronounced within the “*core plus the upstream CTCF motif*” peaks (Additional file 2: Figure S7). A notable exception was the specific increase in signals at the –16 position (reverse strand) and the –25 position (forward strand), both of which correspond to the enhanced CTCF protection signal observed when the upstream motif is present. Overall, this analysis confirms the close association between TOP2B and CTCF and raises the possibility that TOP2B-DNA interactions are affected by the binding of CTCF zinc fingers 9–11 to the upstream CTCF motif.

### Triple sites are enriched at chromosomal domain borders

CTCF and cohesin proteins are key architectural components of the genome that anchor long-range interactions that structure chromosomal domains [62–64]. Multi-species comparisons of chromosomal structure have identified an enrichment of evolutionarily conserved CTCF binding sites at chromosomal domain borders [56]. Given our observation that TOP2B co-localizes with CTCF and cohesin in a specific orientation, we asked whether triple sites are enriched at the boundaries of orientation-specific chromosomal domains.

**Fig. 6:**
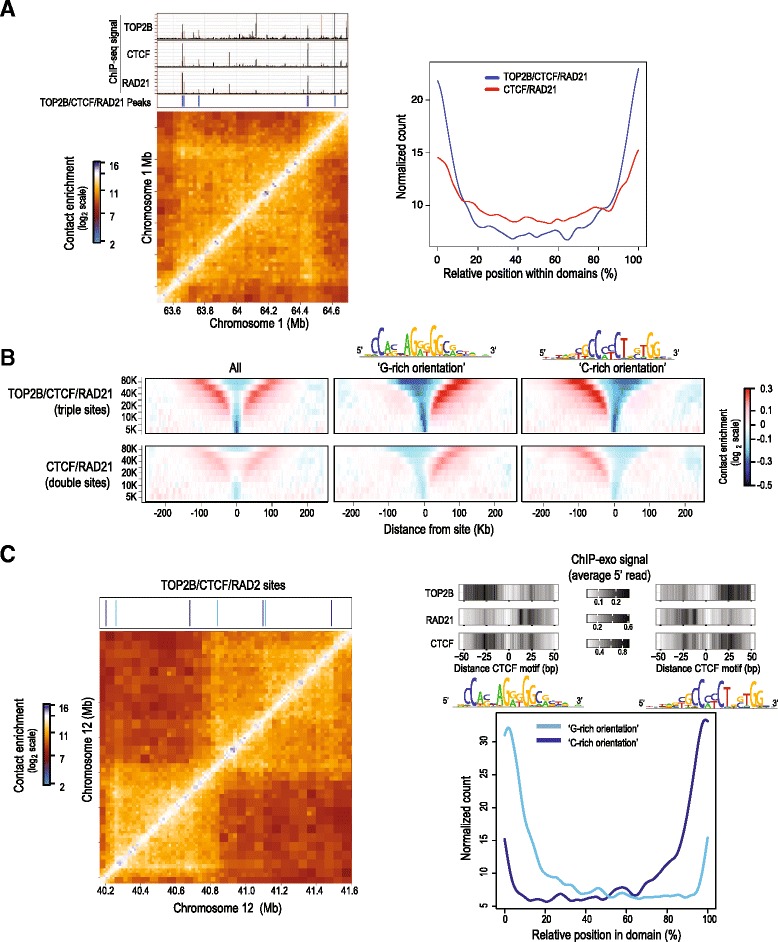
Relationship between TOP2B/CTCF/RAD21 sites and Hi-C structure. **a** TOP2B, CTCF, and RAD21 co-bound sites localize to chromosomal domain borders. ChIP-seq tracks for TOP2B, CTCF, and RAD21 as well as binding sites classified as co-occupied for all three proteins are shown alongside a Hi-C matrix of intra-chromosomal interactions from mouse liver at an example region on chromosome 1 (*left panel*). The genome-wide relative position of TOP2B/CTCF/RAD21 triple sites (*blue*) and CTCF/RAD21 double sites (*red*) within mouse Hi-C domains (*right panel*). **b** Average contact insulation profiles of CTCF/RAD21 sites with (*top panels*) and without (*bottom panels*) TOP2B in mouse Hi-C data. The profiles are also shown after separation based on CTCF motif orientation in the genome (*last two columns*, the CTCF motif is shown above the corresponding column). **c** Binding sites classified as co-occupied for TOP2B/CTCF/RAD21 are shown after separation based on CTCF motif orientation (G-rich orientation, “- strand,” *light blue*; C-rich orientation, “+ strand,” *dark blue*) relative to an example Hi-C region on chromosome 12 (*left panel*). Genome-wide analysis of co-occupied sites, separated based on CTCF motif orientation and their relative position within domains (*right panel*). ChIP-exo profiles centered on CTCF motifs at sites close to domain borders (within 10 % of the full domain size) are also shown

Using recently published mouse liver Hi-C datasets [56], we studied contact insulation profiles [62] centering at triple sites and compared these to CTCF/RAD21 double sites. Triple sites were significantly associated with large-scale chromosomal domain boundaries compared to CTCF/RAD21 sites that lacked TOP2B binding (Fig. 6a). We measured the average contact insulation at triple sites according to the Hi-C data and observed a strong depletion of contacts across these sites at multiple genomic scales, further supporting their localization between two large-scale loops (Fig. 6b). In agreement with our findings on TOP2B/CTCF/RAD21 triple sites from ChIP-seq and ChIP-exo experiments, triple sites showed a higher level of contact insulation compared to double sites, even though the contact insulation patterns for triple and double sites were similar.

Consistent with other reports [54, 56], we found a strong enrichment of CTCF binding to the G-rich motif orientation at the 5′ boundary of domains and a corresponding abundance of CTCF binding to the C-rich motif orientation at the 3′ boundary (Fig. 6c). Together with our ChIP-seq and Chip-exo data, this points to opposite sequential organizations of TOP2B-CTCF-RAD21 sites at the two borders of chromosomal domains with TOP2B positioned external and cohesin internal to the domain loop, which could be involved in the formation and maintenance of large-scale chromatin structures. Given the prominent localization of TOP2B binding at domain boundary CTCF/cohesin sites, we propose that similar to its known function at gene promoters, TOP2B also helps resolve topological constraints around key architectural building blocks of the genome.

### TOP2 proteins facilitate supercoiling at CTCF binding sites

**Fig. 7:**
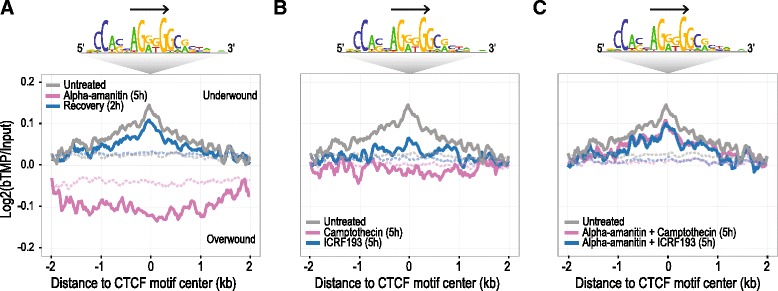
DNA supercoiling around CTCF binding sites. **a** Comparison of DNA supercoiling around CTCF binding sites in untreated human retinal pigment epithelial cells (RPE-1), after 5 h inhibition of RNA polymerase with alpha-amanitin and after 2 h of recovery from inhibition. **b** Changes in DNA supercoiling after 5 h treatment with TOP2 inhibitor ICRF-193 or TOP1 inhibitor camptothecin compared to untreated RPE-1 cells. **c** DNA supercoiling around CTCF sites after simultaneous inhibition of transcription and topoisomerases in RPE-1 cells. Direction of CTCF motif is indicated by the *arrow*. The signal on randomly generated genomic intervals is shown as *dashed lines* and compared with the observed signal (*solid lines*), all *p* values are <10^–16^ (Kolmogorov–Smirnov test). Data and methods used to generate this original analysis were previously published by Naughton et al. [65]

To test the possibility that TOP2 proteins are involved in supercoiling at CTCF sites we reanalyzed the supercoiling domain data of Naughton et al. [65]. Naughton et al. used biotinylated TMP (bTMP) incorporation into the DNA of human retinal pigment epithelial cells to show that chromosome-wide supercoiling, and more specifically supercoiling at TSS, requires the activity of RNA polymerase II as well as topoisomerase I and II proteins [65]. After recapitulating these results at the TSS (Additional file 2: Figure S8), we asked whether supercoiling at CTCF sites also required RNA polymerase II and TOP2 proteins. Indeed, we found that DNA supercoiling at CTCF sites was: (1) lost after treatment with the RNA polymerase II inhibitor alpha-amanitin; (Fig. 7a); (2) is reduced in the presence of TOP2 (ICRF-193) or TOP1 (campothecin) inhibitors (Fig. 7b); and (3) is not affected by topoisomerase inhibition when transcription is inhibited simultaneously (Fig. 7c). In contrast, no specific supercoiling pattern was observed at randomly selected genomic intervals (Fig. 7, dashed lines). Although TOP2 poisons affect both TOP2B and TOP2A, this analysis together with the close association of TOP2B with CTCF raises the possibility that TOP2B can facilitate the remodeling of DNA supercoiling at CTCF sites.

## Discussion

### Extending the TOP2B interactome

We present the first effort to characterize TOP2B’s protein–protein interactome. BioID enabled us to characterize a network of TOP2B proximal protein interactions that are consistent with its localization and function. Most notably, we identified members of the cohesin complex as well as CTCF as significant proximal interacting proteins. From a protein domain perspective, our BioID results show several interactions with zinc finger containing proteins, many of which are novel and relevant to known TOP2B biology (ZNF362, ZNF512, YY1, CTCF, PHF2, and MORC2). For example, MORC ATPases have been shown to be involved in heterochromatin silencing in eukaryotes [66] and following DNA damage, MORC2 can facilitate chromatin remodeling and promote gamma-H2AX induction [67]. As another example, PHF2 is a tumor suppressor that is required for the anticancer effects of doxorubicin in cell lines with active p53 [68]. Finally, CBX8 is part of the polycomb repressive complex 1 (PRC1) [69] involved in the transition from a polycomb-repressed to active chromatin state during ES cell differentiation [70].

TOP2B shuttles between the nucleoplasm and nucleolus and this shuttling involves an RNA interaction with its C-terminal domain [39]. Our BioID results reflect TOP2B’s nucleolar localization. Several of the TOP2B interacting proteins have known nucleolar localization including the: (1) DEAD/DEAH box helicase domain-containing protein DDX18, which is mutated in human AML [71] and implicated as a driver of endocrine resistance in breast cancer cells [72]; (2) DDX31 which helps regulate ribosomal RNA (rRNA) gene transcription in the nucleolus of renal cell carcinomas [73]; and (3) human proteins involved in rRNA processing in the nucleolus (SDAD1 [74] and RRP15 [75]).

TOP2 poisons can affect RNA polymerase I (Pol I) transcription at rDNA loci and this has been shown to involve TOP2A [76]. In addition to TOP2B signals across the coding region of rDNA loci, we detected two distinct TOP2B peaks immediately upstream of the spacer promoter. The spacer promoter associates with Pol I and controls the transcription of an intergenic spacer rRNA that in turn regulates the rDNA promoter in trans [77]. The location of the most distal TOP2B spacer promoter enrichment coincides with activating and repressive histone modifications, TF binding, and Pol I in mouse ES cells [78, 79]. We also observe histone modifications, cohesin subunits, and TFs enriched in this region in mouse liver. The more proximal TOP2B peak, located ~90 bp upstream of the spacer promoter, coincides with a known CTCF binding site that has been implicated in regulating chromatin at the spacer promoter [78, 79]. Consistent with our findings at other CTCF triple sites, RAD21 signal was found just upstream and TOP2B was found downstream of this CTCF motif (which is in its C-rich orientation relative to the rRNA coding region). Given that the spacer promoter region can regulate gene expression in an orientation specific manner in vitro [80], and the importance of CTCF orientation in the regulation of the HOX [81] and protocadherin clusters [82], it will be interesting to test whether CTCF orientation at the spacer promoter affects gene regulation and topology at rDNA loci.

### TOP2B associates with open chromatin and active gene regulatory regions

It is a long-held hypothesis that TOP2 is attracted to a pre-existing combination of DNA sequence and/or chromatin structure [83]. A model where TOP2 binding occurs as a consequence of open chromatin is supported by studies in budding yeast that demonstrated that nucleosome removal enables Top2 binding at specific sites whereas the loss of Top2 does not greatly affect nucleosome positioning [84]. The hypothesis that chromatin structure is a major determinant of TOP2B binding is supported by the following observations: (1) TOP2B occupancy is strongly correlated with DNase I hypersensitive sites; (2) TOP2B occupancy is correlated with allele-specific binding of various TFs; and (3) TOP2B binds to nucleosome-free regions at both proximal and distal promoter sites.

TOP2B-bound open chromatin regions have distinct functional properties. For example, while it is known that CTCF and cohesin play major roles in genomic regulation, CTCF/cohesin sites co-bound by TOP2B showed stronger ChIP-seq signal, are more likely to be evolutionarily conserved, and are enriched at chromatin domain boundaries compared to CTCF/cohesin only sites. HNF4A binding sites co-bound with TOP2B are also enriched for conserved orthologous HNF4A binding. Thus TOP2B co-occupancy not only occurs at chromatin regions that exhibit topological stress induced by genome regulatory function (e.g. promoters and enhancers), but also includes regions of the genome that are fundamentally important for chromatin architecture.

Positioning of TOP2B at promoter, enhancer, and topologically associating domain (TAD) boundaries suggests mechanisms by which tissue-specific DNA damage could be imparted. If TOP2B-induced DSBs are not faithfully re-ligated, adjacent genomic regions are potentially susceptible to genome rearrangements [85], which can give rise to fusion genes and oncogenesis [26, 86]. Interestingly, CTCF/cohesin sites are frequently mutated in cancer [60] and somatic substitutions accumulate immediately adjacent to the CTCF core motif (10–14 bp upstream of center of the G rich CTCF motif). This position overlaps with both the DHS signal and TOP2B ChIP-exo signal near the CTCF motif (Fig. 5c). DNA mutations in cancer cells strongly correlate with DHS sites from the tissue of origin (*r* = 0.8) [87]. While there are many possible explanations for how DNA damage could be biased towards DHS and cohesin/CTCF sites, it is intriguing to speculate whether TOP2B occupancy could influence tissue-specific mutational processes beyond chromosomal rearrangements.

### Spatial organization of TOP2B/CTCF/RAD21 at chromosomal domain borders

CTCF/cohesin sites anchor both chromosomal domains (also known as TADs) [88, 89] as well as local gene loops [62, 64, 90]. Directional CTCF binding is a prominent and evolutionarily conserved feature of chromosomal domain borders [54, 56, 91]. Collectively our proteomics and ChIP data clearly reveal the close association between TOP2B, cohesin, and CTCF, raising the question of whether TOP2B contributes to the long-range contact networks anchored by these architectural proteins. Indeed, using Hi-C datasets from mouse liver samples [56], we show an enrichment of triple sites at borders of chromosomal domains. Our ChIP-seq and ChIP-exo analyses (Fig. 5) show a striking spatial organization of triple sites relative to the G-rich CTCF motif. This organization places TOP2B at the base of the domain loop, with cohesin being inside the domain loop.

TADs contain supercoiling domains whose borders are also enriched for CTCF binding sites [65]. Based on TOP2B/CTCF protein–protein and protein–DNA interactions and our analysis of DNA supercoiling at CTCF binding sites in the presence of TOP2 poisons [65] (Fig. 7), we suggest that TOP2B can facilitate DNA supercoiling at CTCF binding sites in a transcription-dependent manner.

## Conclusions

We only have a basic understanding of how the ubiquitously expressed TOP2B selectively regulates gene expression in vivo. Detailed information about protein–protein and DNA–protein interactions of TOP2B is important for understanding its role in development, rapid gene expression, and chemotherapeutic responses. We identified cohesin and several other chromatin proteins that are in close proximity to TOP2B in vivo. We demonstrated that TOP2B binding occurs at evolutionarily conserved TF binding sites and topological domain boundaries. The prevalent occupancy of TOP2B at conserved gene regulatory and chromatin architectural regions indicates that TOP2B is intrinsically positioned to function at actively utilized points of genome control.

## Methods

### Construct and stable HeLa cell culture generation

Construct for *TOP2B* gene was generated via Gateway cloning into pDEST 5′ BirA*-FLAG-pcDNA5-FRT-TO. *TOP2B* (accession #NM_001068) was cloned into pDONR223 entry vector using pooled human cDNA and sequence verified. TOP2B bait protein tagged with a N-terminal BirA*-FLAG tag (*n* = 6 replicates) was stably expressed in Flp-In T-REx HeLa cells as described [31]. Parental Flp-In T-REx HeLa cells (*n* = 6) and stable cells expressing BirA*-FLAG fused to a green fluorescent protein (GFP; *n* = 3) or to a nuclear localization sequence (NLS; *n* = 3) were used as negative controls for the BioID experiments and processed in parallel to the TOP2B bait expressing cells. Stable cell lines were grown to 80 % confluence before expression was induced via 1 μg/mL tetracycline and biotinylation by the addition of 40 μM biotin for 24 h. Subsequently, cells were washed and harvested in ice-cold PBS and frozen at −80 °C until purification.

### Proximity biotinylation coupled with mass-spectrometry

Equal quantities of starting material were used for each BioID experiment. HeLa cell pellets were thawed in 1.5 mL ice-cold modified RIPA buffer (50 mM Tris–HCl (pH 7.4), 150 mM NaCl, 1 % NP-40, 1 mM MgCl2, 1 mM EGTA, 0.1 % SDS, and 0.4 % sodium deoxcycholate). Sigma protease inhibitor cocktail (P8340, 1:500) and PMSF (1 mM) were added prior to use. The lysates were sonicated at 4 °C using three 5 s bursts at 35 % amplitude with 3 s pauses. Samples were treated with 250 U of TurboNuclease (BioVision) for 15 min followed by removal of insoluble material by centrifugation at 20,000 g. The supernatant was transferred to a new tube and 30 μL of pre-washed streptavidin-sepharose bead slurry (GE Healthcare, Cat 17-5113-01) was added. Biotinylated proteins were captured on the beads for 4 h at 4 °C with rotation. The beads were washed once with 1 mL of 2 % SDS in 25 mM Tris (pH 7.4), once with 1 mL of standard RIPA buffer, once with 1 mL of TNNE (50 mM Tris-HCl (pH 7.4), 150 mM NaCl, 0.1 % NP-40, 1 mM EDTA). Lastly, the beads were washed three times with 1 mL of 50 mM ammonium bicarbonate, pH 8.0 (ABC). Following the final wash, the beads were pelleted and any excess liquid was aspirated off. The proteins captured on the beads were resuspended in ABC, reduced with 5 mM DTT at 50 °C for 30 min, and alkylated using 50 mM iodoacetamide for 20 min at room temperature in the dark. The proteins were digested overnight with gentle rotation at 37 °C with 1 μg of trypsin (Sigma, T7575) in a total volume of 50 μL. In the following morning, an additional 0.5 μg of trypsin was added for an additional incubation of 2–4 h. The beads were pelleted and the peptide supernatant was transferred to a fresh tube. The beads were rinsed twice with 75 μL HPLC-grade water and the wash fraction was combined with the supernatant. The peptide solution was acidified with 50 % formic acid to a final concentration of 5 % and the samples were dried in a centrifugal evaporator. Tryptic peptides were re-suspended in 15 μL 5 % formic acid and stored at −80 °C until analyzed by mass spectrometry. Mass spectrometry and data analysis were carried out as described previously [31]. Briefly, using an Eksigent Autosampler, 5 μL of the tryptic peptides were loaded at 400 nl/min on to a 75 μm × 12 cm fused silica capillary tubing packed with 3 μm-C18 (ReproSil-PurC18-AQ). Peptides were subjected to nano-LC-ESI-MS/MS, using a 90 min reversed phase (5–35 % acetonitrile, 0.1 % formic acid) buffer gradient, delivered at 200 nl/min and analyzed on a TripleTOF 5600 (AB SCIEX). The instrument performed a 250 ms MS1 TOF survey scan from 400–1300 Da followed by 20 100 ms MS2 candidate ion scans from 100–2000 Da in high sensitivity mode.

### MS data analysis

Raw mass spectrometry files were stored, searched, and analyzed using the ProHits laboratory information management system (LIMS) [92]. The WIFF data files were converted to MGF format using WIFF2MGF and subsequently converted to an mzML format using ProteoWizard (3.0.4468) [93] and the AB SCIEX MS Data Converter (V1.3 beta). The mzML files were searched using Mascot (v2.3.02) and Comet (2014.02 rev.2) [94], essentially as described by Lambert et al. [31].

Briefly, the spectra were searched against a total of 72,230 proteins consisting of the NCBI human and adenovirus complements of the RefSeq database (v57, forward and reverse sequences), supplemented with “common contaminants” from the Max Planck Institute (http://maxquant.org) and the Global Proteome Machine (GPM; http://www.thegpm.org/crap/index.html).

The database parameters were set to search for tryptic cleavages, allowing up to two missed cleavage sites per peptide, MS1 mass tolerance of 40 ppm with charges of 2+ to 4+, and an MS2 mass tolerance of +/− 0.15 amu. Carbamidomethylation on cysteine was selected as a fixed modification and deamidated asparagine/glutamine and oxidized methionine were selected as variable modifications.

The results from each search engine were analyzed through TPP (the Trans-Proteomic Pipeline, v4.7) [95] via the iProphet pipeline [96]. SAINTexpress version 3.3 [97] was used with default parameters to calculate statistical significance of each potential protein–protein interaction relative to control samples. Only proteins identified with minimally two unique peptides ions and a minimum iProphet probability of 0.95 were considered. The bait replicates (*n* = 6) were compressed to three samples, meaning that after SAINTexpress was run on each sample individually, the three highest SAINTexpress scores were averaged for the final scoring and Bayesian FDR assessment. To increase the stringency in the identification of true positives, the 12 controls were also compressed to four; in this case, the compression is performed before running SAINTexpress by selecting the four highest spectral counts for each prey protein for modeling [98]. All control samples were deposited in the Contaminant Repository for Affinity Purification (www.crapome.org) [98] and assigned the following identifiers: CC831, CC834, CC835, CC838, CC842 (BirA*-FLAG-GFP), CC837, CC840, CC841 (BirA*-FLAG-NLS) and CC832, CC833, CC836, CC839 (parental cells). For western blot confirmation of BioID results, we carried out the BioID protocol as described above. After the last wash, the streptavidin beads were re-suspended in 60 uL of Laemmli sample buffer containing 200 uM Biotin and boiled for 5 min then resolved on 8 % SDS-PAGE. Twenty microliters of sample was loaded for each western blot lane. The membranes were probed for RAD21 (ab992, Abcam), STAG1 (ab4457, Abcam), and CTCF (07-729, Millipore) and developed using BioRad Gel Doc XR system. For each immunoprecipitation the equal amount of material collected from 10-cm tissue culture dishes was used.

### Functional enrichment and data visualization

Significantly enriched pathways were computed with the g:Profiler software [99], using ordered enrichment analysis on significance-ranked proteins and custom filtering (3–1000 proteins in the pathway, at least two interacting proteins per pathway, FDR corrected *q* <0.05; Additional file 1). Biological processes from Gene Ontology, pathways from the KEGG and Reactome databases, and protein complexes from the CORUM database were included in the analysis and other functional annotations were filtered. Pathways were visualized using Cytoscape software using the Enrichment Map plugin [100].

### Mouse tissue material

#### Mouse liver material for ChIP-seq and ChIP-exo

Post-mortem liver material from male C57BL/6 × A/J mice (aged ~6–8 weeks) were kindly provided by Dr. Duncan Odom (Cambridge Research Institute). C57BL/6J mice (aged ~6–8 weeks) post-mortem livers used for ChIP-seq and ChIP-exo were kindly provided by Dr. Jayne Danska. Fresh liver tissue was fixed for 20 min in 1 % formaldehyde as described previously [101].

### ChIP-seq

ChIP-seq experiments were performed as described previously [101]. The following antibodies were used: anti-TOP2B (sc-13059, Santa Cruz Biotechnology; *n* = 5), anti-CTCF (07-729, Millipore; *n* = 4), anti-RAD21 (ab992, Abcam; *n* = 2), anti-H3K36me3 (13C9 monoclonal kindly provided by Hitoshi Kimura; n = 1), anti-H3K4me3 (ab8580, Abcam; *n* = 1), anti-H3K4me2 (07-030, Millipore; *n* = 1). The DNA was end-repaired, dA-tailed, ligated to the sequencing adapters, PCR amplified by 16 cycles using multiplexing index primers (NebNext), size selected (200–350 bp, PippinPrep, Sage Science), quantified with 2100 Bioanalyzer (Agilent), and 50 bp reads were sequenced with the HiSeq2500 (Illumina).

### ChIP-exo

We used an Illumina ChIP-exo protocol [102] adapted from the original protocol described by [59, 102]. ChIP was performed as described previously until and including the RIPA buffer washes at step 38 [101]. Seven micrograms of antibody against the TOP2B, CTCF, and RAD21 was used for each biological replicate. Two biological replicates for TOP2B and RAD21 ChIP-exo experiments and one biological replicate for CTCF were used for downstream analysis.

### Public data resources

Publicly available datasets used in this study include: mouse liver ChIP-seq of multiple liver expressed regulatory factors and histone modifications (Data accession: E-MTAB-941) [40], mouse liver ChIP-seq of histone modifications from mouse ENCODE (Data accession: GSM1000153, GSM1000140) [41], mouse liver DHS-seq (dccAccession: wgEncodeEM002906) [103], mouse liver RNA-seq (Data accession: GSM1015152) [104], and nucleosome occupancy data (Data accession: GSM717558) [45], CTCF ChIP-seq data of mouse, human, rat, and dog liver tissues (Data accession: E-MTAB-437) [42], and supercoiling profiling data (Data accession: E-GEOD-43450) [65]. CTCF binding regions in multiple adult mouse tissues were obtained from the mouse ENCODE database [41]. Published CTCF peaks from human retinal pigment epithelial cells (HRPEpiC) [105] were obtained from (GSM749673). Data quality control results and a full list of links to processed files are available (Additional file 3).

### Read alignment and quality control of ChIP-seq data

ChIP-seq sequencing reads were trimmed to 36 bp and aligned to the reference mouse genome assembly (mm9, GRCh37) available at UCSC genome browser database using the Burrows-Wheeler Aligner (http://bio-bwa.sourceforge.net/) [106] with default parameters. To remove sequencing and mapping artifacts, we discarded all reads mapping to regions of the ENCODE blacklist (https://sites.google.com/site/anshulkundaje/projects/blacklists). Only uniquely mapped reads were used for further analysis.

Quality of the datasets processed from the raw sequencing reads was assessed following the ENCODE ChIP-seq guidelines [107]. Quality control information with references and accession numbers are available (Additional file 3). Peak calling for quality control was performed using MACS2 software [108] without input and with significance cutoff *q* = 0.01.

Validation of TOP2B antibodies was performed using RIME (rapid immunoprecipitation mass spectrometry of endogenous proteins) [109]. RIME assay was performed as previously described using mouse liver tissue from 8-week-old mice. Fifteen micrograms of antibody (TOP2B sc-13059 (*n* = 2) or IgG sc-2027 (*n* = 1)) was used for each ChIP (Additional file 2: Figure S9).

### Peak calling

The reads of biological replicates and corresponding input samples were merged for peak calling. Read counts and peak numbers used in our analyses are listed in Additional file 2: Table S1. Peaks from ChIP-seq data were called using the MACS2 method [108] with the significance cutoff of *q* = 0.01 and fold change cutoff of 5. The “–keep-dup” option was set to “all” to keep duplicated reads. For histone modifications and RNA polymerase II binding, peaks were called with additional “—broad –broad-cutoff 0.05” options. To compare ChIP-seq and ChIP-exo peaks, the SWEMBL (www.ebi.ac.uk/~swilder/SWEMBL) peak caller was used with parameter “-R 0.005.”

### Genomic annotation of TOP2B binding

The genomic distribution of TOP2B binding was annotated using the cis-regulatory element annotation system (CEAS) [110]. *p* values were calculated with R using the one-sided binomial test. The overlap of TOP2B ChIP-seq binding sites with binding sites of other factors was calculated using bedtools intersect [110]. The significance of the overlaps was accessed using Genomic Association Test (GAT) [111] with 1000 simulations. All *q* values were smaller then 10^–3^.

Using pairwise Pearson correlation coefficients as a distance measurement, we clustered multiple ChIP-seq experiments using hierarchical clustering and visualized the result as a heatmap with the R bioconductor package DiffBind [112]. Peak regions for all factors were first merged to a consensus peak set. Read counts per million mapped reads (RPKM) of each factor across this consensus peak set were computed.

### Profiling TOP2B ChIP-seq signal over gene bodies

Processed RNA-seq gene expression values for mouse liver (GSM1015152) [104] were log transformed and separated into three groups based on the mean ± SD of the values (high, medium, and low expression). TOP2B ChIP-seq signal (RPM, normalized to regions length) was plotted across gene bodies of the three groups of genes using the NGSplot package [113].

### Profiling TOP2B ChIP-seq signal on rDNA

To analyze the binding of TOP2B and other factors at rDNA loci, we constructed a customized mouse genome with the single rDNA repeat sequence included as an extra chromosome. Mouse rDNA sequence and structure were obtained from GenBank accession no. BK000964. Reads were aligned to this customized genome using bwa with default parameters. Only uniquely mappable reads were used for downstream analysis. Reads were extended to 150 bp prior to plotting. After normalizing to the number of mapped reads for each ChIP-seq and input experiment, input reads were subtracted from ChIP-seq reads at each base pair of the rDNA repeat. Plotting was performed using R package “Sushi” [114]. Mappability data was obtained from Zentner et al. and displayed as a heatmap below the tracks with black representing 100 % mappability [78].

### Comparing ChIP-seq with DHS, gene density and GC content

Aligned Dnase I Digital Genomic Footprinting (DGF) data for mouse liver were obtained from the ENCODE database (see Additional file 3). Only uniquely mapped reads were used and ENCODE blacklist regions were excluded from the genome. For both the DGF data and ChIP-seq data, numbers of reads in every 10 kb across the whole genome were counted. Pairwise Spearman correlation between DGF and ChIP-seq data was calculated based on these values. Similarly, gene density and GC content was calculated for all 10 kb windows across the genome. For visualization, values larger than 99.5 % percentiles were removed and a smoothing spline curve was fit to the data using R. Finally, smoothed values were scaled and centered on 0 before plotting.

### Nucleosome occupancy profile

Coordinates of nucleosomes previously mapped in mouse liver were used [45]. Nucleosome regions mapped to the mouse reference genome mm8 were lifted over to mm9 using liftOver tool and chain files from UCSC database [115]. ChIP-seq peak summits of each factor were separated into two categories: proximal (< ±1 kb) and distal (> ±1 kb) relative to the TSS of transcripts annotated in Ensembl database (build 37). Each summit was extended to 1.5 kb towards both 5′ and 3′ directions. The extended proximal summit regions were ordered based on the direction of the nearest transcript so that the direction of transcription always pointed to the right. The nucleosome positions were mapped to the extended regions around the summits and the average number of nucleosomes mapped to each position was plotted separately for proximal and distal binding regions of each factor.

### De novo motif discovery

Regions 50 bp upstream and downstream of TOP2B peak summits were extracted and used for de novo motif discovery (Fig. 3c) using the RSAT peak-motifs method with default settings [116].

### Detection and analysis of triple sites

At least 1 bp overlapping binding regions of TOP2B, CTCF, and RAD21 were determined with bedtools merge function [117]. Merged regions were then annotated according to the co-occupying factors. Numbers of overlapping peaks of these three factors are shown in the three-way Venn diagram. The merged regions co-occupied by the three factors were referred as “triple sites.” For each factor, the binding intensity (RPKM of each peak region) of the original peaks annotated by different overlapping patterns was calculated and plotted as boxplots. *p* values were calculated with Wilcoxon rank sum test followed by multiple testing corrections using the Benjamin–Hochberg method.

### Comparing CTCF peaks across multiple tissues

All mouse liver CTCF peaks identified in this study were overlapped with the CTCF peaks identified in 14 tissues of 8-week-old adult mice (bone marrow, bone marrow derived microphage, cerebellum, cortex, heart, kidney, liver, lung, MEF, olfactory bulb, small intestine, spleen, testis, thymus) by the Ren lab as part of the mouse ENCODE release (Additional file 3). Each peak was annotated by the tissues in which it overlapped with least one ENCODE CTCF peaks. If the peaks were shared in more than seven tissues, it would be defined as “constitutive” across tissues. Fisher’s exact test was applied to determine if the triple site CTCF peaks are more likely to be constitutive compared to other CTCF peaks.

### Evolutionary conservation of triple sites

CTCF peak regions were scanned for the CTCF core motif using the RSAT matrix-scan method [116] with the command “matrix-scan -v 1 -quick -i -m -matrix_format transfac -origin start -bginput -markov 1 -2str -uth pval 0.0001 -return pval.” A window of 150 bp upstream and downstream of the motif center was then extracted and ordered based on the motif direction. Average GERP score of each bp around the motif summits were then calculated and plotted. We used the mouse GERP score track available in the UCSC Genome Browser Database: ftp://hgdownload.cse.ucsc.edu/gbdb/mm9/bbi/All_mm9_RS.bw.

Conservation analysis was based on the detection of the CTCF ChIP-seq peaks found in mouse in the orthologous regions in human, rat, and dog using Ensembl Compara API (build 70). In order to match the genome assembly used in Ensembl 70, CTCF peaks identified in mm9 were lifted over to mm10 using the liftOver tool and the chain file provided by UCSC Genome Browser Database [115]. Triple sites, CTCF-RAD21 double sites, and CTCF singleton sites were divided into three phylogenetic categories: Mouse only; shared in mouse and rat (Rodents only); and shared in mouse and/or rat and at least in one non-rodent species (dog, human) (Beyond rodents). Fisher’s exact test was used to test if numbers of deeply conserved sites (Beyond rodents) were significantly different between different categories of CTCF peaks.

### B2 SINE element analysis

The Repeatmasker method (Smit AFA, Hubley R: RepeatModeler Open-1.0.2008-2010; http://www.repeatmasker.org) was run on the genome sequences of CTCF peaks. Only peaks having the B2 SINE repeat overlapping their peak summits were included in the analysis.

### Directionality analysis of triple sites

Triple site regions were scanned for the CTCF core motif using the RSAT matrix-scan method [116] as described above. If multiple motifs were found within one peak region, only the motif with the highest weight was used. The genomic distances between CTCF core motif center and nearest peak summits of CTCF, RAD21, and TOP2B in triple sites were calculated. The distributions of distances were visualized as violin plots before and after orienting all CTCF motifs to the G-rich direction. Wilcoxon rank sum tests were used to compare between the distances before and after orientating CTCF motif. The orders of CTCF motif center, RAD21 and TOP2B peak summits were listed according to the distances calculated before and after correcting for CTCF motif direction, and plotted as bar plots. Fisher’s exact tests were used to compare the likelihood of observing peaks with certain ordering before and after orientating CTCF motif.

### ChIP-exo analysis

Sequencing reads for ChIP-exo experiments were aligned without trimming. All reads were used in the analysis. ChIP-seq of the same factors was performed in parallel for comparison. We applied the SWEMBL peak caller algorithm that is sensitive for point-source data (http://www.ebi.ac.uk/~swilder/SWEMBL/). Peak overlaps were performed and plotted using DiffBind [112]. The ChIP-exo Profiler method [118] was used to generate TOP2B, CTCF and RAD21 ChIP-exo-seq and DGF sequencing read profiles around the CTCF binding motif. Specifically, CTCF binding regions identified previously by the triple site analysis were scanned with the CTCF core motif. Next, flanking regions of 50 bp upstream and downstream from the center of CTCF core motif were retrieved and ordered based on the motif direction. Regions with less than ten mapped ChIP-exo reads were discarded. To calculate the average 5′ coverage at each nucleotide position around the CTCF motif, numbers of first 5′ nucleotides of ChIP-exo reads mapped to each position were counted and divided by total number of regions. Reads from forward and reverse strands were mapped separately. To control for the effect of sequence composition, the CTCF core motif was permuted ten times using RSAT permute-matrix function [119]. Motif scanning and read profiling were also performed for each of the permuted matrices to build a random background (shown as shaded polygons on Fig. 5e).

### Allele-specific binding analysis

ChIP-seq data from an F1 mouse (C57BL/6 female × A/J male) were used to investigate the allele-specific binding preferences of TOP2B at locations bound by specific TFs in mouse liver. Single nucleotide polymorphisms (SNPs) obtained from the Sanger Mouse Genomes Project version 2 were used to acquire a list of SNPs between the A/J and reference (C57BL6/J) genomes [120]. Aligned reads were processed with the WASP pipeline [121] to remove reads with potential alignment bias between parental genomes and remove duplicate reads. Reads overlapping SNP positions (allelic reads) were then separated based on their parent of origin using the ALEA pipeline [122]. In doing so, we considered only reads that overlapped an informative allele and that could be mapped unambiguously to one parent.

Considering only the overlapping regions of the TOP2B and the TF in question, we counted the number of allelic reads mapped to each parental genome to determine an allele frequency. Peaks showing significantly biased allelic read distribution (binomial *p* <0.05) were annotated based on the mouse strain possessing TF-preferred allele. A one-sided Wilcoxon ranked sum test was used to compare the allele frequency of each factor between allelic biased regions and non-biased regions.

### Hi-C data analysis

The Hi-C data were obtained from Vietri Rudan et al. (GSE65126) [56]. Please refer to that work for details about the Hi-C libraries, normalization methods, and contact insulation analysis. The relative distribution of CTCF within TADs was calculated as the distance of each CTCF site from the center of its domain. Half the size of the domain was added to convert it to a measure of distance from the edge of the domain and this value was subsequently divided by the size of the domain.

### DNA supercoiling analysis

Processed files containing DNA microarray probe intensities were obtained from ArrayExpress (E-GEOD-43450). Data were processed as previously described with normalized bTMP incorporation calculated as log_2_(bTMP cell /bTMP input) – log2(bTMP genomic DNA – bTMP input) [65]. GENCODE hg19 gene annotation was used to extract TSS positions. CTCF sites from human retinal pigment epithelial cells (HRPEpiC) [105] were used. For each probe, the nearest TSS or CTCF motif center within a CTCF peak was found, the distance from the probe to the feature was calculated with regard to the direction of transcription or the CTCF motif. Distances were binned by 100 bp and median intensity of the binned probes was calculated. Finally, a rolling mean method with a sliding window of size = 10, step = 2 was applied prior to plotting data. Same number of genomic regions was randomly generated and probe intensity around these regions were calculated in the same manner. The random selection was performed 10 times and an average value was used as the random background, which is plotted as a dashed line with corresponding colors. The Kolmogorov–Smirnov test was used to compare between the random background and the actual profile (dashed versus solid lines of same colors in each panel of Fig. 7 and Additional file 2: Figure S6. All *p* values were smaller than 10^–16^.

## Additional files

Additional file 1: TOP2B interactome, and statistically over-represented biological processes in the TOP2B interactome. (XLSX 158 kb) Additional file 2: Supplementary tables and figures. (XLSX 193 kb) Additional file 3: Overview of the genomic datasets used. (DOCX 2034 kb) Additional file 4: List of de novo motif discovery results. (PDF 363 kb)
