# Supplementary material for: Topoisomerase II beta interacts with cohesin and CTCF at topological domain borders
Source: Genome Biol. 2016 Aug 31;17(1):182. doi: 10.1186/s13059-016-1043-8 (PMC5006368; doi:10.1186/s13059-016-1043-8)
Supplement: Additional file 3: — Overview of the genomic datasets used. (DOCX 2034 kb) [file 13059_2016_1043_MOESM3_ESM.docx]

**Additional file 3, supplementary tables and figures**

**Topoisomerase II beta interacts with cohesin and CTCF at topological domain borders**

Liis Uusküla-Reimand†, Huayun Hou†, Payman Samavarchi-Tehrani, Matteo Vietri Rudan, Minggao Liang, Alejandra Medina-Rivera, Hisham Mohammed, Dominic Schmidt, Petra Schwalie, Edwin J. Young, Jüri Reimand, Suzana Hadjur, Anne-Claude Gingras, and Michael D. Wilson^*^

**Table S1. Quality control of ChIP-seq experiments.** Summary of the factors and mouse liver samples used in this study. Sample ID column contains the library IDs for biological replicates. The reads of biological replicates were merged and filtered (#reads), and peaks were called for merged libraries (#peaks) using either MACS2 (M) or SWEMBL (S) peak callers. See **Methods** and **Additional file 2** for further details.

| **Factor** | **Assay** | **Library ID** | **Input ID** | **#reads** | **#peaks** | **Peak caller** |
| --- | --- | --- | --- | --- | --- | --- |
| TOP2B | ChIP | do178,do349,do71 | do297,do28 | 48877272 | 53723 | M |
| STAG1 | ChIP | do172,do867 | do279,do28,do566 | 34538586 | 40834 | M |
| STAG2 | ChIP | do868 | do566 | 22320221 | 51798 | M |
| GABPA | ChIP | do200 | do297,do28 | 13302736 | 16252 | M |
| H3K4eme1 | ChIP | do201 | do297,do28 | 5625682 | 39219 | M |
| HNF4A | ChIP | do27 | do297,do28 | 10481815 | 47382 | M |
| RAD21 | ChIP | do303 | do297,do28 | 13462171 | 70205 | M |
| H3K79me2 | ChIP | do319 | do297,do28 | 14572436 | 65837 | M |
| H3k4me3 | ChIP | do350,do607 | do279,do28,do566 | 27556569 | 16854 | M |
| H3K36me3 | ChIP | do352 | do297,do28 | 9663399 | 48066 | M |
| ONECUT1 | ChIP | do40,do41 | do297,do28 | 36244183 | 48737 | M |
| FOXA1 | ChIP | do463,do466 | do566 | 29848151 | 46593 | M |
| CTCF | ChIP | do464,do781 | do566 | 38583122 | 49196 | M |
| RNAP2 | ChIP | do465,do468 | do566 | 32055850 | 25151 | M |
| HNF1A | ChIP | do62 | do297,do28 | 15227779 | 22159 | M |
| CEBPA | ChIP | do70,do73 | do566 | 24295320 | 56390 | M |
| CBP | ChIP | do978 | do566 | 12694440 | 32995 | M |
| YY1 | ChIP | do1500, do1596 | do566 | 48097748 | 21538 | M |
| H3K4me2 | ChIP | WL9 | do566 | 22728129 | 30203 | M |
| H3K27Ac | ChIP | wgLicr_H3k27Ac | wgLicr_Input | 24911074 | 33417 | M |
| H3K9Ac | ChIP | wgLicr_H3k09Ac | wgLicr_Input | 33284594 | 26966 | M |
| CTCF | ChIP | do39 , do302 | do297,do28 | 29335015 | 66972 | M |
| CTCF | ChIP | do506 , do778 | do205,do648 | 43111674 | 58431 | M |
| CTCF | ChIP | do804 ,do807 | do810,do811 | 59535079 | 59003 | M |
| CTCF | ChIP | do301, do780 | do100,do107,do261 | 31025076 | 58985 | M |
| CTCF | ChIP | WL122 , WL16 | WL123, WL132 | 30271008 | 41926 | S |
| ONECUT1 | ChIP | WL120 , WL133 | WL123, WL132 | 31041751 | 59747 | S |
| RAD21 | ChIP | WL121 , WL134 | WL123, WL132 | 24451073 | 48291 | S |
| TOP2B | ChIP | WL124 , WL135 | WL123, WL132 | 32918357 | 11308 | S |
| CTCF | ChIP-exo | WL314 | WL123, WL132 | 15470697 | 36394 | S |
| RAD21 | ChIP-exo | WL312, WL315 | WL123, WL132 | 32527929 | 8907 | S |
| TOP2B | ChIP-exo | WL310 , WL313 | WL123, WL132 | 32063825 | 2891 | S |

**Table S2.** Genome wide correlations between ChIP-seq signal intensities (RPM: reads per million reads) of studied factors, DNase I hypersensitivity sites (DHS), gene density, and genomic GC content in mouse liver. Spearman correlation coefficients of the genome-wide signal are shown (all *p*-values are < 2.2e-16).

| **Factor** | **DHS** | **Gene density** | **GC content** |
| --- | --- | --- | --- |
| TOP2B | 0.806 | 0.367 | 0.513 |
| FOXA1 | 0.742 | 0.332 | 0.536 |
| STAG2 | 0.728 | 0.313 | 0.551 |
| H3K4me1 | 0.727 | 0.345 | 0.485 |
| STAG1 | 0.721 | 0.310 | 0.531 |
| RAD21 | 0.712 | 0.307 | 0.517 |
| ONECUT1 | 0.683 | 0.277 | 0.329 |
| H3K79me2 | 0.671 | 0.494 | 0.441 |
| H3K9ac | 0.661 | 0.383 | 0.634 |
| HNF4A | 0.660 | 0.270 | 0.357 |
| RNAP2 | 0.652 | 0.448 | 0.460 |
| H3K36me3 | 0.638 | 0.499 | 0.464 |
| CBP | 0.637 | 0.286 | 0.469 |
| H3K27ac | 0.632 | 0.313 | 0.457 |
| CTCF | 0.622 | 0.298 | 0.497 |
| H3K4me2 | 0.616 | 0.327 | 0.515 |
| CEBPA | 0.606 | 0.292 | 0.473 |
| EP300 | 0.598 | 0.207 | 0.223 |
| HNF1A | 0.583 | 0.276 | 0.474 |
| GABPA | 0.538 | 0.211 | 0.229 |
| H3K27me3 | 0.487 | 0.245 | 0.386 |

**Table S3.** Cross species comparison of CTCF binding. CTCF binding sites grouped as TOP2B/CTCF/RAD21 triple sites, CTCF/RAD21 and CTCF/TOP2B double sites, and CTCF only sites are further separated by their conservation levels: conserved in mouse only, mouse and rat only and beyond rodents (mouse plus at least one non-rodent (human, macaque, dog)). The numbers of CTCF sites that contain rodent-specific B2 SINE elements are shown in parentheses.

|  | **Total CTCF binding sites (with B2 SINE)** | **Mouse only CTCF sites**  **(with B2 SINE)** | **Mouse and rat only CTCF sites**  **(with B2 SINE)** | **Beyond Rodents CTCF sites**  **(with B2 SINE)** |
| --- | --- | --- | --- | --- |
| TOP2B/CTCF/RAD21 | 20,381 (1,349) | 6,385 (762) | 4,765 (565) | 9,231 (22) |
| CTCF/RAD21 | 20,049 (6,656) | 11,372 (4,828) | 4,546 (1,750) | 4,131 (78) |
| CTCF/TOP2B | 394 (12) | 263 (7) | 34 (1) | 97 (4) |
| CTCF | 8,301 (3,002) | 5,708 (2,410) | 1,390 (543) | 1,203 (49) |

**Table S4.** Cross species comparison of HNF4A binding. HNF4A binding sites grouped as TOP2B/HNF4A/RAD21 triple sites, HNF4A/RAD21 and HNF4A/TOP2B double sites, and HNF4A only sites are further separated by their conservation levels: conserved in mouse only, mouse and rat only and beyond rodents (mouse plus at least one non-rodent (human, macaque, dog)).

|  | **Total HNF4A binding sites** | **Mouse only HNF4A sites** | **Mouse and rat only HNF4A sites** | **Beyond Rodents HNF4A sites** |
| --- | --- | --- | --- | --- |
| TOP2B/HNF4A/RAD21 | 15,831 | 8,562 | 3,146 | 4,123 |
| HNF4A/RAD21 | 3.865 | 2,684 | 596 | 585 |
| HNF4A/TOP2B | 7,145 | 4,058 | 1,305 | 1,782 |
| HNF4A | 20,538 | 15,788 | 2,483 | 2,267 |


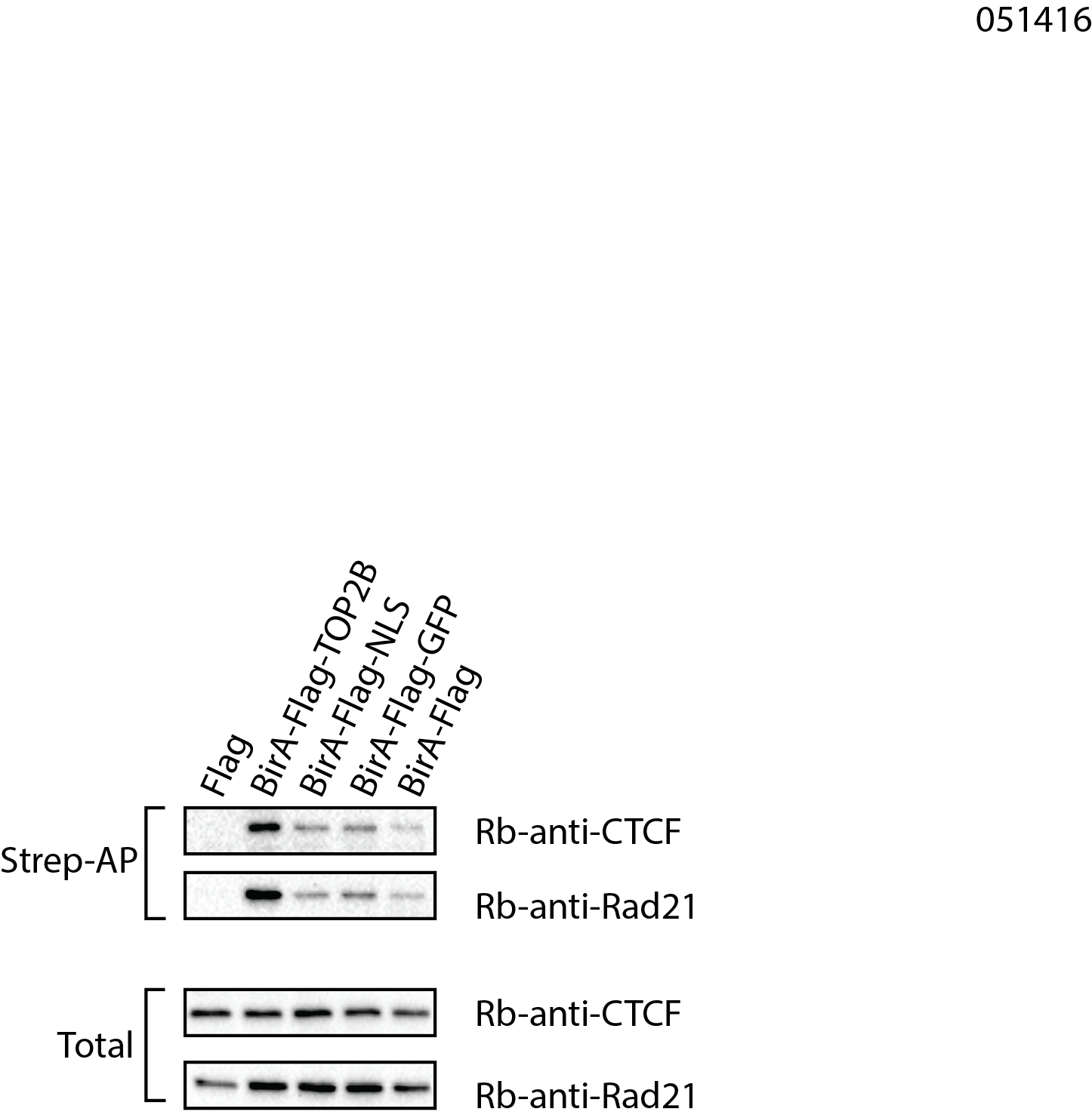


**Fig. S1. Western blot confirmation of TOP2B BioID hits for CTCF and RAD21.** The Flp-In TREx HeLa cell lines were induced with tetracycline and treated with biotin as described in the methods section. The BirA*-Flag fused to TOP2B (lane 2), nuclear localization signal (NLS, lane 3), green fluorescent protein (GFP, lane 4), or BirA* by itself (lane 5), as well as the parental FLp-In TREx HeLa cells (Lane 1) were used in affinity purification comparison. Following the BioID protocol described in the methods, samples were boiled in sample buffer containing 2mM biotin. 10% of the supernatant was resolved by SDS-PAGE. Equal amount of starting material was used for the streptavidin pulldown for each bait. Western blotting of 10% streptavidin pull downs was performed using anti-CTCF antibody (row 1) and anti-RAD21 antibody (row 2). Western blotting of 5% total cell lysates from each experiment prior to streptavidin pulldown is shown in rows 3 and 4.


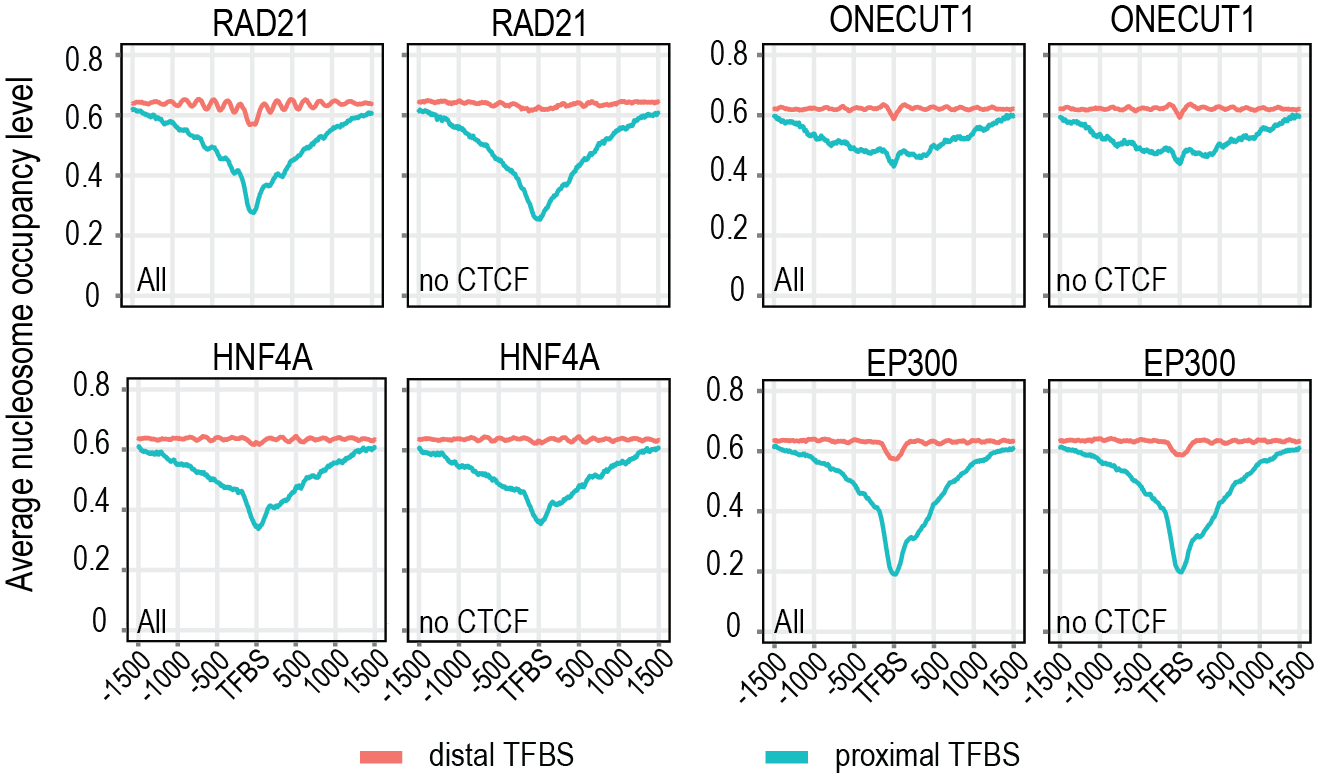


**Fig. S2. Nucleosome positioning around binding sites of RAD21, HNF4A, ONECUT1 and EP300.** Nucleosome occupancy profiles for all RAD21, HNF4A, ONECUT1, and EP300 peaks (All) centered on the peak summit are shown for proximal (≤ 1kb away from promoters, red lines) and distal peaks (>1kb away from promoters, blue lines). Profiles for RAD21, ONECUT1, HNF4A and EP300 peaks that do not overlap with CTCF peaks (no CTCF) are also shown.


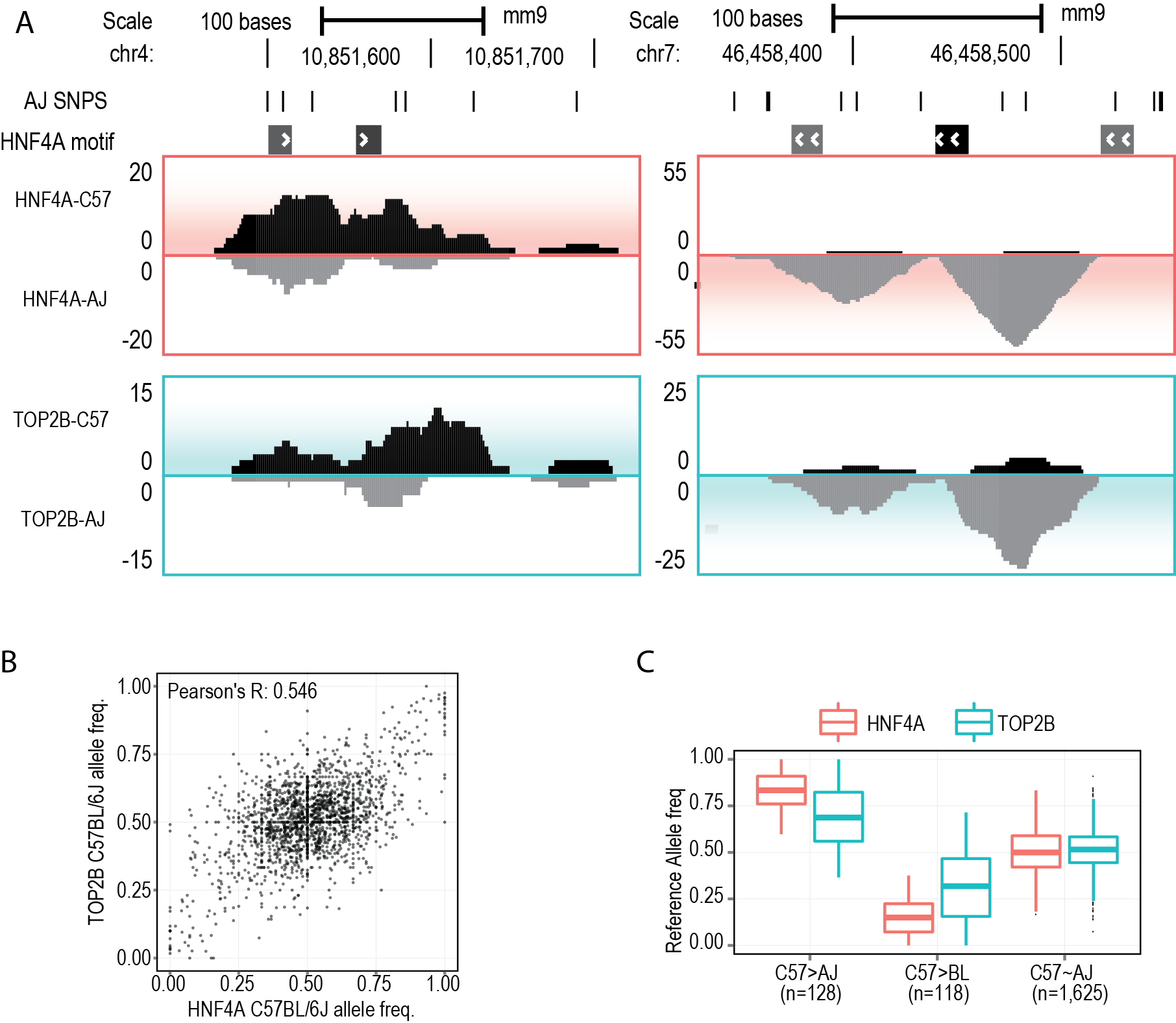


**Fig. S3. Allele-specific binding of TOP2B and HNF4A is correlated. a** Genome browser view of example HNF4A bound regions that show allele-specific bias towards the C57BL/6J genome (left panel) and A/J genome (right panel) (y-axis, number of allelic reads). **b** Correlation of C57BL/6J allele frequencies for HNF4A (x-axis) versus TOP2B (y-axis) at TOP2B/HNF4A co-occupied regions. **c** TOP2B (blue) and HNF4A (red) C57BL/6J allele frequencies (blue) at TOP2B/HNF4A co-occupied regions, categorized based on HNF4A allelic binding preference (see Methods). C57>A/J indicates sites with HNF4A preference for the C57BL/6J allele, A/J>C57 indicates preference for the A/J allele, and C57~A/J indicates sites with no significant allelic-specific bias. The TOP2B allelic frequencies for both C57BL/6J and A/J enriched HNF4A binding sites were significantly different than the TOP2B allelic frequencies in C57~A/J category (*p=*2.98e-33 and *p*=7.15e-32, respectively; One-sided Wilcoxon rank-sum test).


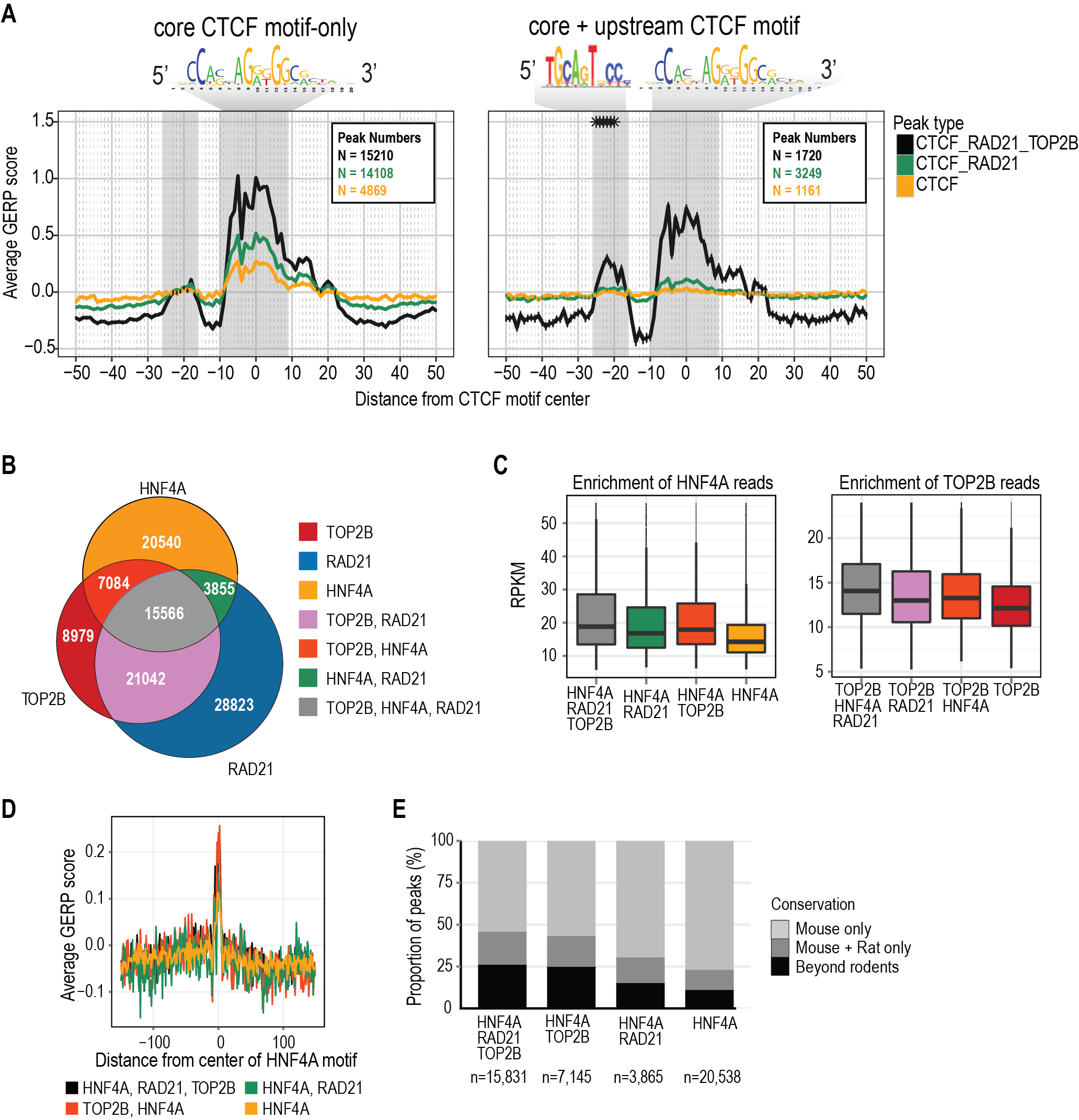


**Fig. S4.** **Genomic features of combinatorial TOP2B binding.** **a.** Comparison of DNA constraint between CTCF peaks with and without the upstream motif. CTCF peaks were first separated by the presence of the CTCF upstream motif. We then divided the CTCF peaks into: CTCF, TOP2B, RAD21 (“triple sites”, black line), CTCF, RAD21 (“double sites”, green line) and CTCF single sites (yellow line). The number of peaks in different categories are shown within each plot. The GERP profile for core CTCF motif only peaks is shown on the left and the GERP profile for core plus upstream CTCF motif peaks is shown on the right. Locations where the GERP score in triple sites with the upstream motif are significantly different than triple sites without the upstream motif are indicated with an asterisk (Student’s one-tailed t test, adjusted p-value < 0.05). **b** Overlap of TOP2B, HNF4A, and RAD21 ChIP-seq binding regions defines seven different categories of peaks. **c** Comparison of HNF4A and TOP2B ChIP-seq reads (RPKM) for peaks in each of the categories defined in **b.** Outliers (>95^th^ percentile) are not shown. **d** DNA sequence constraint of different HNF4A peaks as determined by average GERP score (y-axis). Peaks were oriented based on the direction of HNF4A motif. **e** Cross species comparison of mouse HNF4A peaks with HNF4A ChIP-seq peaks mapped in human, macaque, rat and dog. Stacked bar plots show the proportion of peaks based on the degree of conservation (mouse only, mouse and rat only, and mouse plus one non-rodent (beyond rodents)).


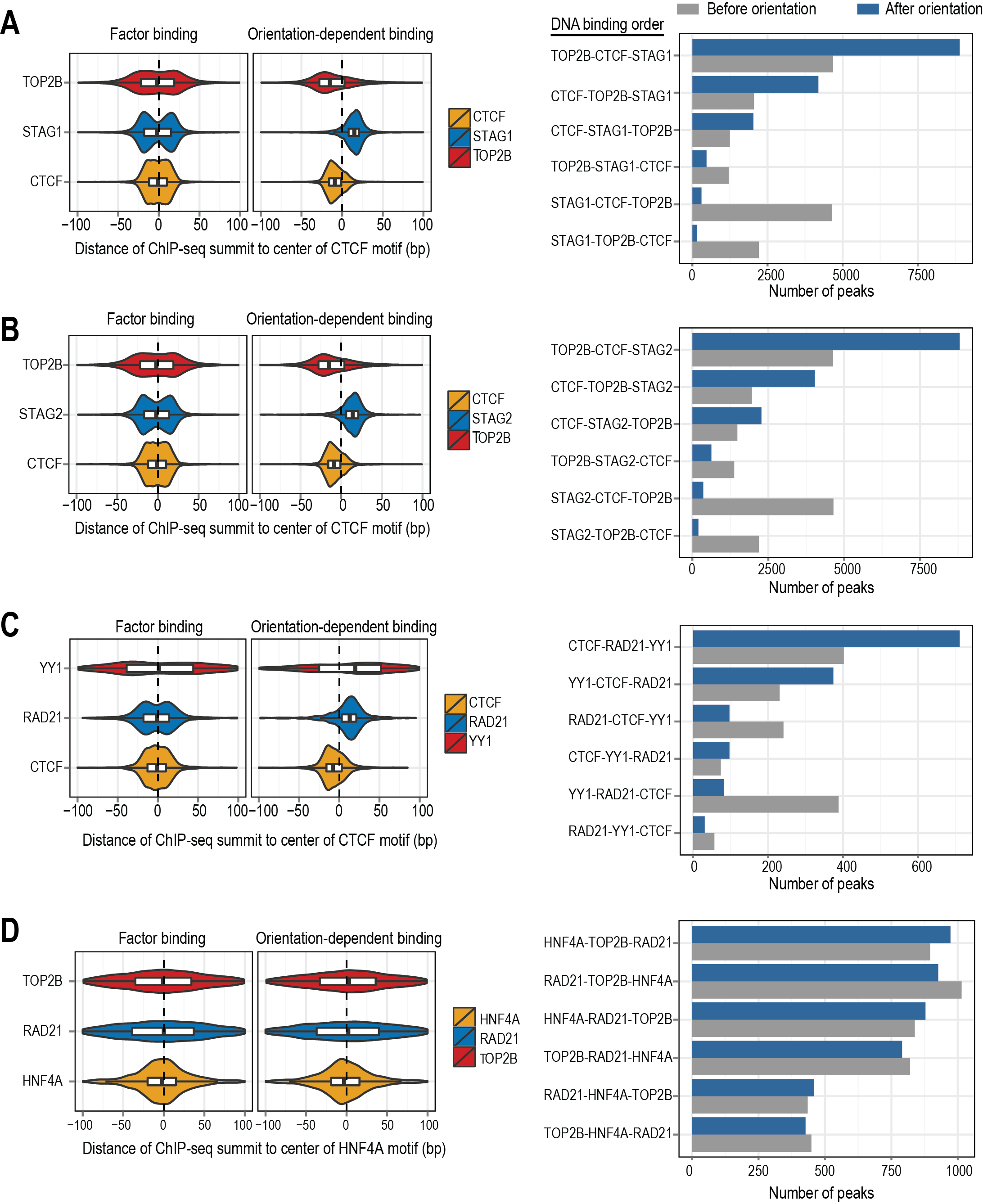


**Fig. S5. Spatial organization of cohesin subunits (STAG1, STAG2, RAD21), TOP2B and YY1 binding around CTCF or HNF4A motifs. Left panels**: Distribution of ChIP-seq peak summits relative to the center of CTCF core motif (**a-c**) or HNF4A motif (**d**). Distribution is shown before and after ordering the peaks according to the orientation of the CTCF or HNF4A motifs. **Right panels**: number of triple sites with different orders of factor binding before (grey) and after (blue) accounting for CTCF (**a-c**) or HNF4A (**d**) motif orientation.


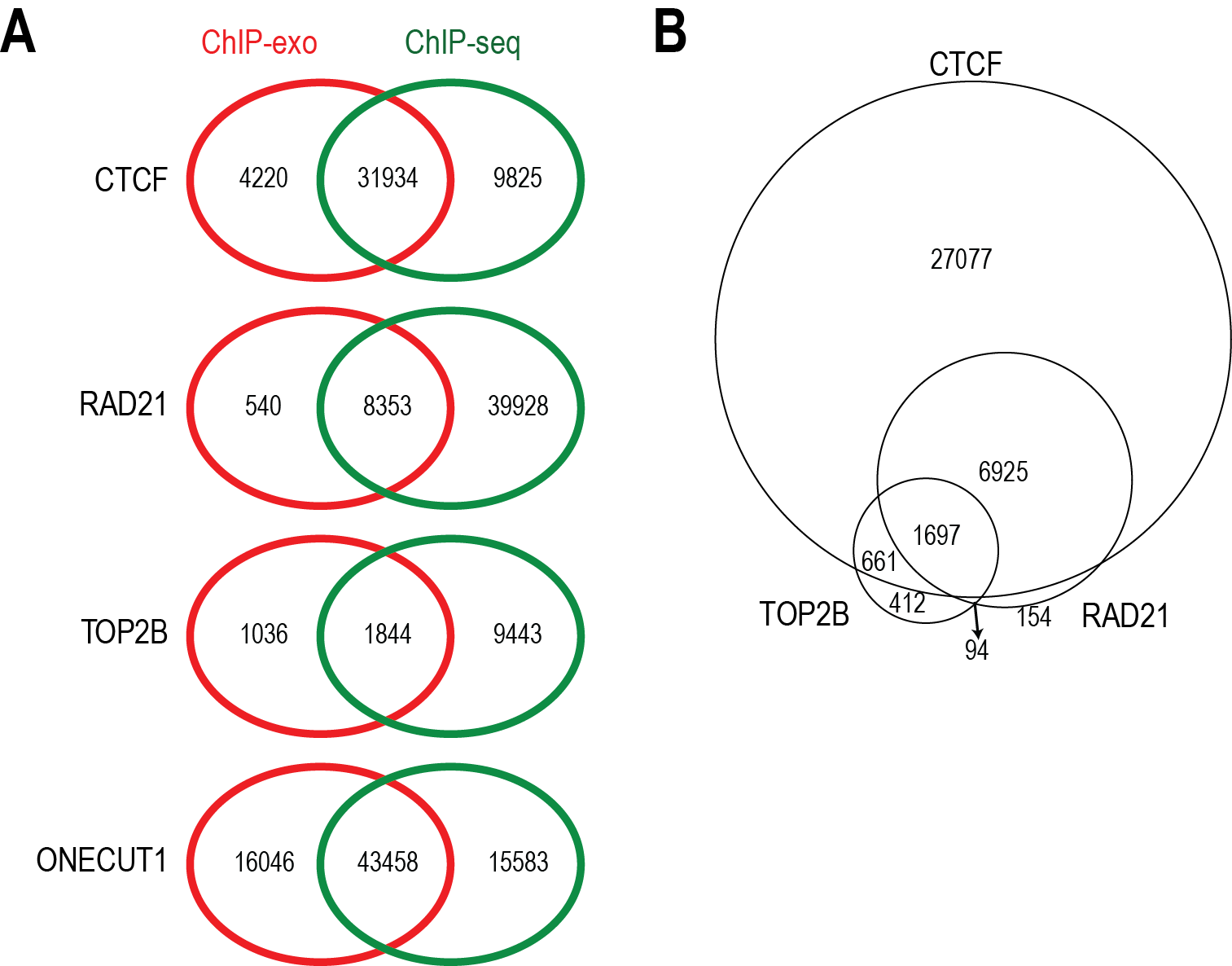


**Fig. S6. Overlap of ChIP-exo and ChIP-seq peaks.** **a** Overlap of CTCF, RAD21, TOP2B and HNF6 ChIP-exo and ChIP-seq peaks called using SWEMBL. **b** TOP2B ChIP-exo peaks extensively overlap with CTCF and RAD21 ChIP-exo peaks.


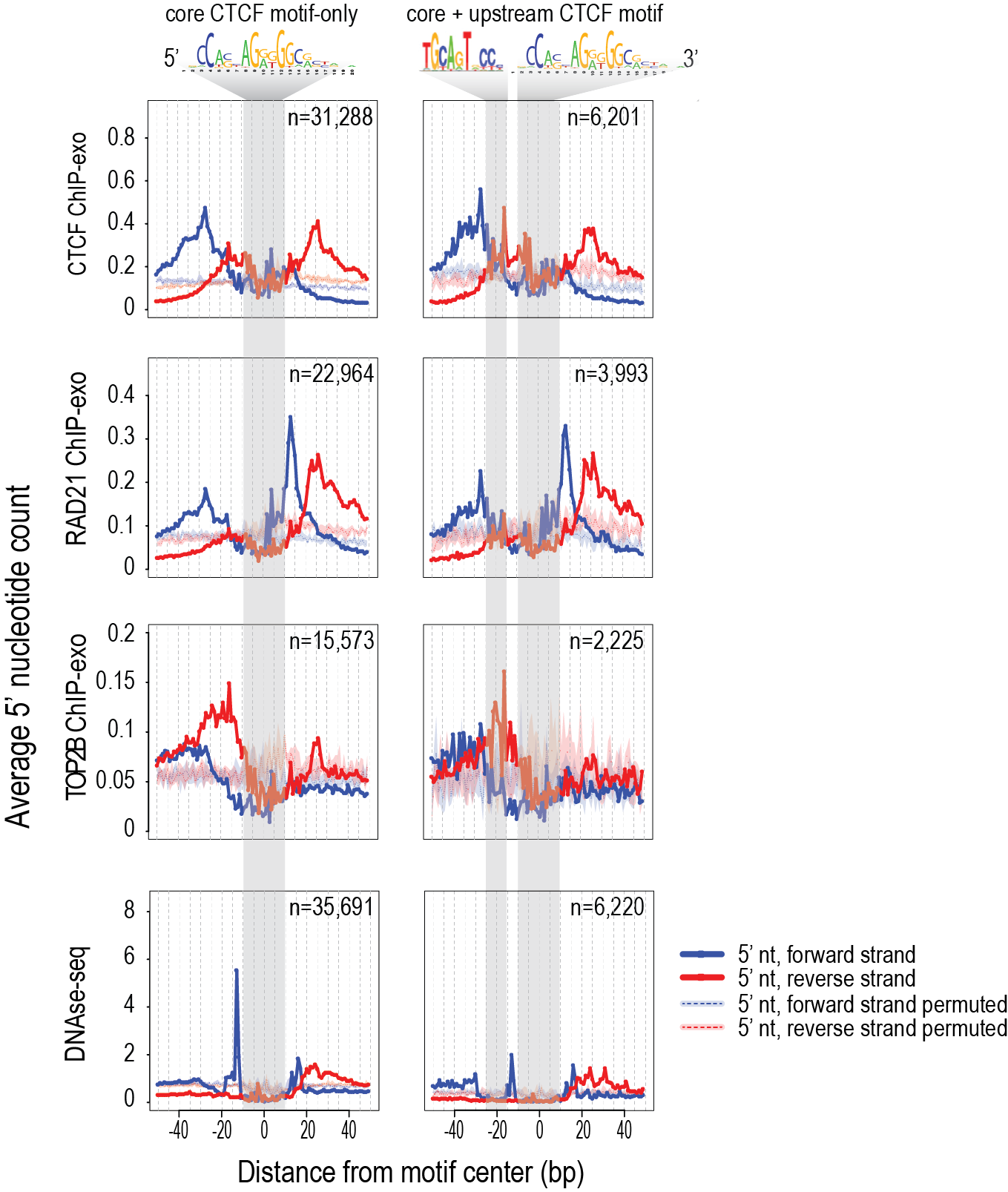


**Fig. S7.** Comparison of TOP2B ChIP-exo protection signal at CTCF peaks with and without the upstream CTCF motif (‘*core plus the upstream CTCF motif*’ and *‘core CTCF motif-only’* respectively). Average 5’ nucleotide coverage (blue: forward strand; red: reverse strand) is plotted for peaks with only the CTCF core motif (left panel), and peaks with both the core and upstream motif (right panel). Average 5’ nucleotide coverage DNAse I hypersensitivity profiling reads obtained for mouse liver are shown to illustrate that CTCF peaks with the upstream motif have a fundamentally distinct DNA accessibility profile. The grey shading illustrates the location of the core CTCF motif in both panels. In the right panel the leftmost grey shading is flanked by the -25 and -16 positions that show stronger CTCF ChIP-exo protection signals in the presence of the upstream CTCF motif.

**
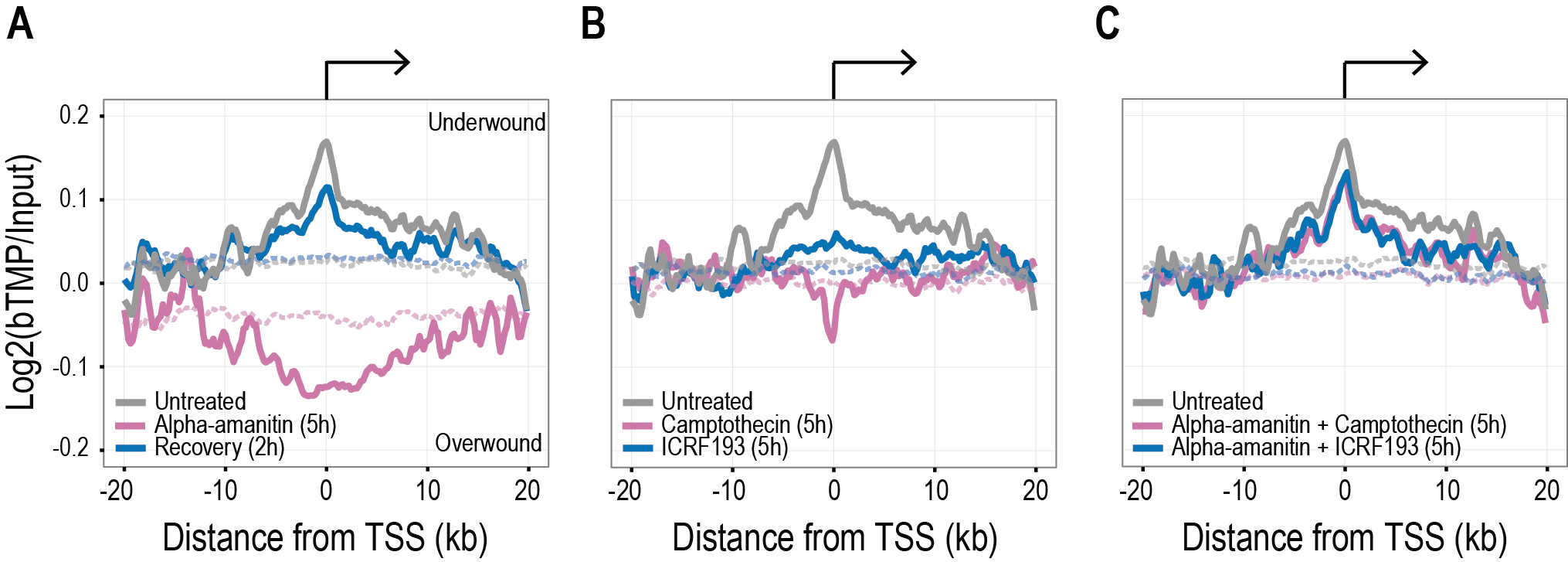
**

**Fig. S8. DNA supercoiling around transcription start sites (TSSs) in human retinal pigment epithelial cells (RPE1) a** Comparison of DNA supercoiling around TSSs in untreated cells (grey), after 5h inhibition of RNA polymerase (pink) and after 2h of recovery from inhibition (blue). **b** Changes in DNA supercoiling after 5h treatment with TOP2 inhibitor ICRF-193 (blue) or TOP1 inhibitor camptothecin (pink) compared to untreated **RPE1** cells (grey). **c** DNA supercoiling around TSSs after simultaneous inhibition of transcription and topoisomerases in **RPE1** cells (pink: camptothecin treatment; blue: ICRF-193 treatment) compared to untreated cells (grey). Direction of transcription is indicated by arrow. Signal on randomly generated genomic intervals are shown as dashed lines and compared with the observed signal (solid lines), all *p* values are < 10^-16^ (K-S test).


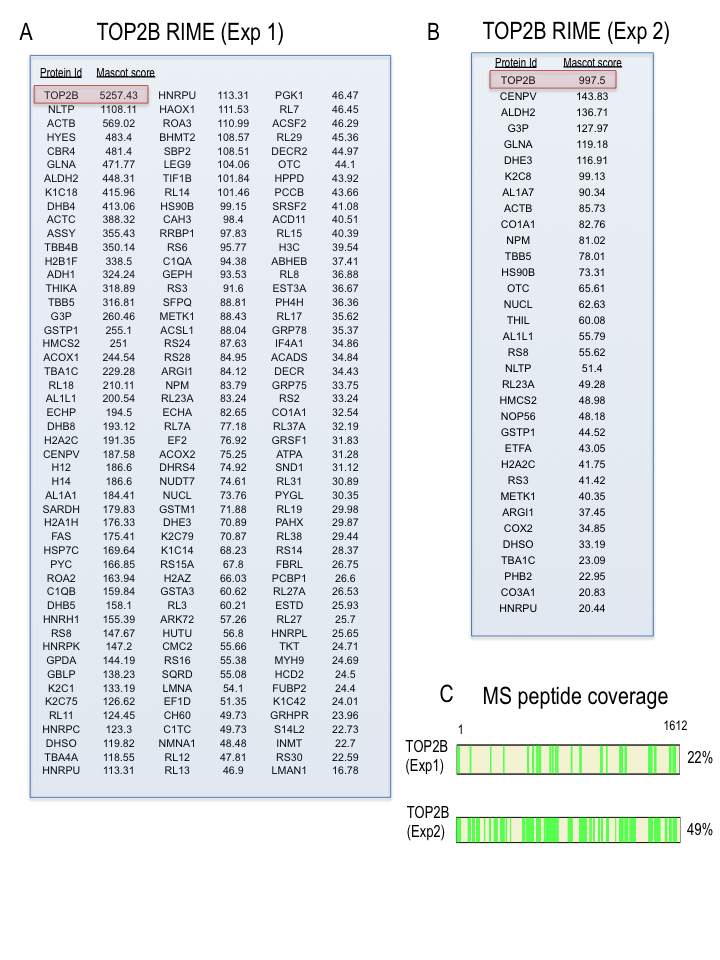


**Fig. S9. Proteomic validation of TOP2B antibody**. Purification of endogenous TOP2B and Mass spectrometry of associated proteins was performed using the RIME assay. Results for TOP2B antibody sc-13059 that identifies TOP2B along with other potential interactions for (**a**) biological replicate 1 and (**b**) biological replicate 2. High Mascot scores for TOP2B suggest good specificity for both antibodies. Results were filtered against a non-specific IgG control. **c** Mass Spectrometry peptide coverage (highlighted in green) of TOP2B for both RIME experiments.
