# Supplementary material for: Topoisomerase II beta interacts with cohesin and CTCF at topological domain borders
Source: Genome Biol. 2016 Aug 31;17(1):182. doi: 10.1186/s13059-016-1043-8 (PMC5006368; doi:10.1186/s13059-016-1043-8)
Supplement: Additional file 4: — List of de novo motif discovery results. (PDF 363 kb) [file 13059_2016_1043_MOESM4_ESM.pdf]

Motif discovery results using all peaks

Motif	Logo	3 Top hits in databases
oligos_6nt_mkv4_m1	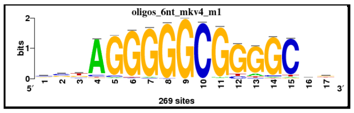	versus jaspar_core_vertebrates: SP1, SP2, KLF5,
oligos_6nt_mkv4_m2	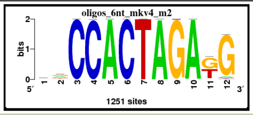	versus jaspar_core_vertebrates: CTCF, ZNF354C,
oligos_6nt_mkv4_m3	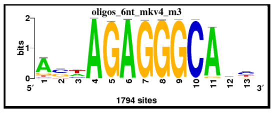	versus jaspar_core_vertebrates: CTCF,
oligos_6nt_mkv4_m4	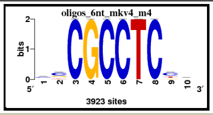	versus jaspar_core_vertebrates: SP2, SP1,
oligos_6nt_mkv4_m5	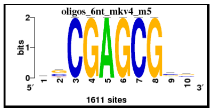	versus jaspar_core_vertebrates: no match
positions_6nt_m1	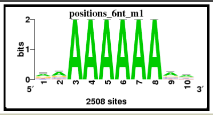	versus jaspar_core_vertebrates: Foxd3,
positions_6nt_m2	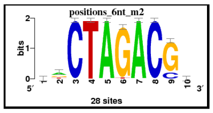	versus jaspar_core_vertebrates: no match
positions_6nt_m3	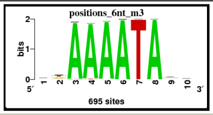	versus jaspar_core_vertebrates: MEF2C, MEF2A,
positions_6nt_m4	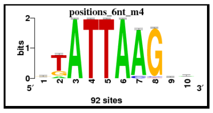	versus jaspar_core_vertebrates: NKX3-1, HNF1B, Prrx2,
positions_6nt_m5	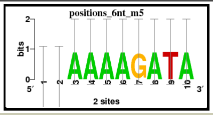	versus jaspar_core_vertebrates: Mecom, Gata1, Gata4,
oligos_7nt_mkv5_m1	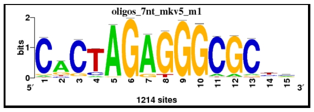	versus jaspar_core_vertebrates: CTCF,
oligos_7nt_mkv5_m2	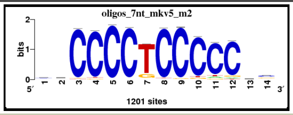	versus jaspar_core_vertebrates: SP1, EGR1, KLF5,
oligos_7nt_mkv5_m3	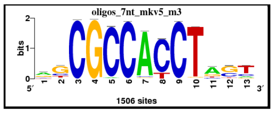	versus jaspar_core_vertebrates: CTCF,
oligos_7nt_mkv5_m4	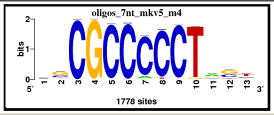	versus jaspar_core_vertebrates: CTCF, SP2, INSM1,

oligos_7nt_mkv5_m5	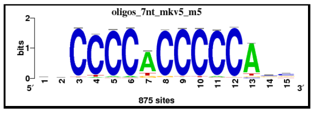	versus jaspar_core_vertebrates: KLF5, SP1, EGR1,
positions_7nt_m1	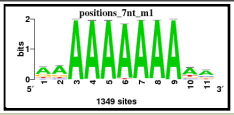	versus jaspar_core_vertebrates: Foxd3,
positions_7nt_m2	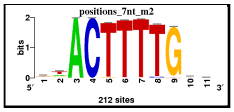	versus jaspar_core_vertebrates: Sox3,
positions_7nt_m3	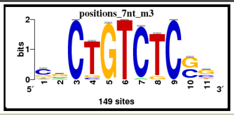	versus jaspar_core_vertebrates: no match
oligos_8nt_mkv6_m1	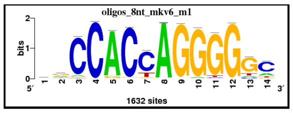	versus jaspar_core_vertebrates: CTCF,
oligos_8nt_mkv6_m2	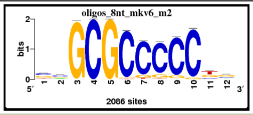	versus jaspar_core_vertebrates: INSM1, CTCF,
oligos_8nt_mkv6_m3	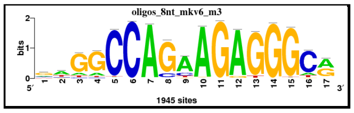	versus jaspar_core_vertebrates: CTCF,
oligos_8nt_mkv6_m4	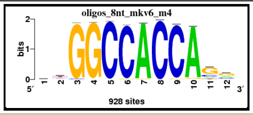	versus jaspar_core_vertebrates: CTCF,
oligos_8nt_mkv6_m5	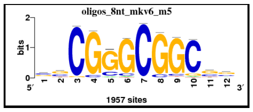	versus jaspar_core_vertebrates: no match
positions_8nt	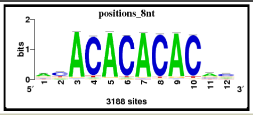	versus jaspar_core_vertebrates: no match

Motif discovery results using peaks not overlapping Ctcf peaks

Motif	Logo	3 Top hits in databases
oligos_6nt_mkv4_m1	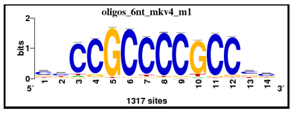	versus jaspar_core_vertebrates: SP1, KLF5, SP2,
oligos_6nt_mkv4_m2	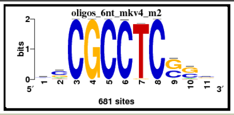	versus jaspar_core_vertebrates: SP2,
oligos_6nt_mkv4_m3	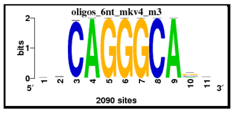	versus jaspar_core_vertebrates: TFAP2C, ESR1, TFAP2A,
oligos_6nt_mkv4_m4	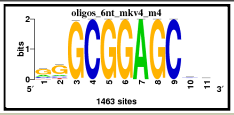	versus jaspar_core_vertebrates: SP1, SP2,
oligos_6nt_mkv4_m5	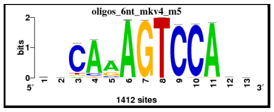	versus jaspar_core_vertebrates: HNF4A, HNF4G, Rxra,
positions_6nt_m1	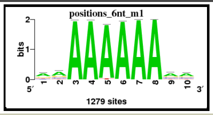	versus jaspar_core_vertebrates: Foxd3,
positions_6nt_m2	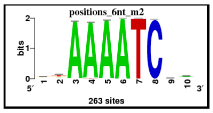	versus jaspar_core_vertebrates: Gfi1b, Gfi1, NFATC2,
positions_6nt_m3	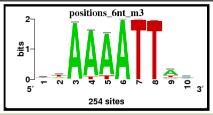	versus jaspar_core_vertebrates: Prrx2, HOXA5, Pdx1,
oligos_7nt_mkv5_m1	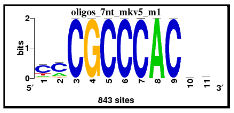	versus jaspar_core_vertebrates: EGR2, EGR1, E2F4,
oligos_7nt_mkv5_m2	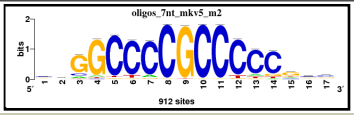	versus jaspar_core_vertebrates: SP2, SP1, KLF5,
oligos_7nt_mkv5_m3	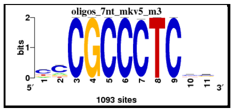	versus jaspar_core_vertebrates: SP2, KLF5, EGR1,
oligos_7nt_mkv5_m4	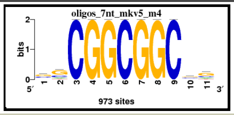	versus jaspar_core_vertebrates: no match
oligos_7nt_mkv5_m5	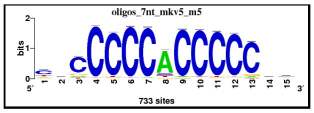	versus jaspar_core_vertebrates: SP1, EGR1, KLF5,
oligos_8nt_mkv6_m1	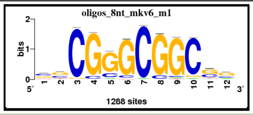	versus jaspar_core_vertebrates: no match

oligos_8nt_mkv6_m2	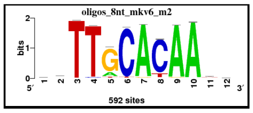	<u>versus jaspar_core_vertebrates:</u> CEBPA, CEBPB, SOX10,
oligos_8nt_mkv6_m3	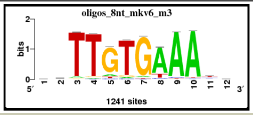	<u>versus jaspar_core_vertebrates:</u> CEBPA, SOX10, CEBPB,
oligos_8nt_mkv6_m4	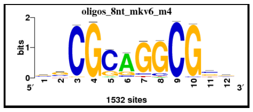	<u>versus jaspar_core_vertebrates:</u> NRF1, E2F1,
oligos_8nt_mkv6_m5	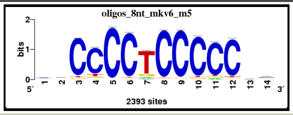	<u>versus jaspar_core_vertebrates:</u> SP1, EGR1, KLF5,
